# Development of a Desorption Electrospray Ionization–Multiple-Reaction-Monitoring Mass Spectrometry (DESI-MRM) Workflow for Spatially Mapping Oxylipins in Pulmonary Tissue

**DOI:** 10.1021/acs.analchem.4c02350

**Published:** 2024-10-26

**Authors:** Matthew J. Smith, Mu Nie, Mikael Adner, Jesper Säfholm, Craig E. Wheelock

**Affiliations:** † Unit of Integrative Metabolomics, Institute of Environmental Medicine, Karolinska Institutet, SE-171 77 Stockholm, Sweden; ‡ Department of Respiratory Medicine and Allergy, Karolinska University Hospital, SE-171 64 Stockholm, Sweden; § Experimental Asthma and Allergy Research Unit, Institute of Environmental Medicine, Karolinska Institutet, SE-171 77 Stockholm, Sweden

## Abstract

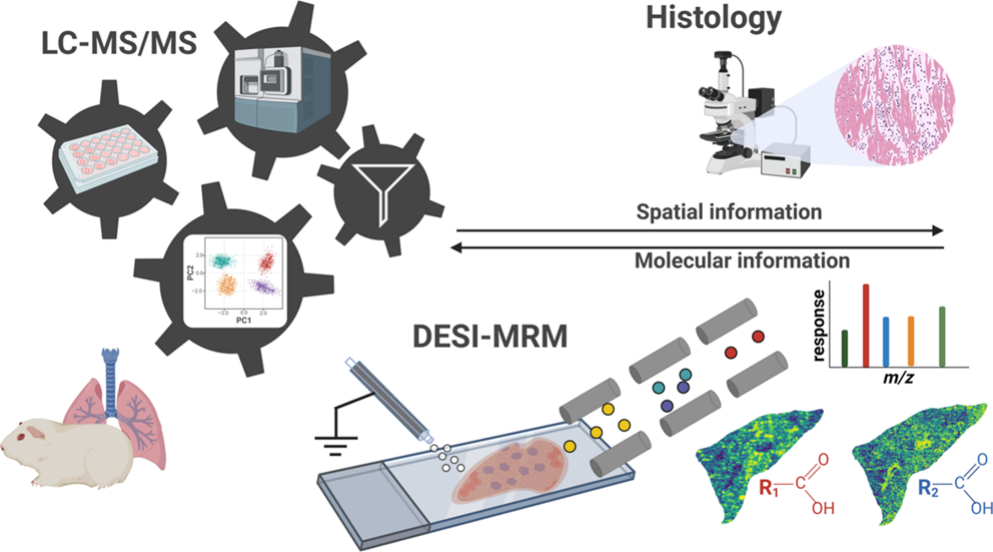

Oxylipins are a class of low-abundance lipids formed via oxygenation of fatty acids. These compounds include potent signaling molecules (e.g., octadecanoids, eicosanoids) that can exert essential functions in the pathophysiology of inflammatory diseases including asthma. While some oxylipin signaling cascades have been unraveled using LC-MS/MS-based methods, measurements in homogenate samples do not represent the spatial heterogeneity of lipid metabolism. Mass spectrometry imaging (MSI) directly detects analytes from a surface, which enables spatial mapping of oxylipin biosynthesis and migration within the tissue. MSI has lacked the sensitivity to routinely detect low-abundance oxylipins; however, new multiple-reaction-monitoring (MRM)-based MSI technologies show increased sensitivity. In this study, we developed a workflow to apply desorption electrospray ionization coupled to a triple quadrupole mass spectrometer (DESI-MRM) to spatially map oxylipins in pulmonary tissue. The targeted MSI workflow screened guinea pig lung extracts using LC-MS/MS to filter oxylipin targets based on their detectability by DESI-MRM. A panel of 5 oxylipins was then selected for DESI-MRM imaging derived from arachidonic acid (TXB_2_, 11-HETE, 12-HETE), linoleic acid (12,13-DiHOME), and α-linolenic acid (16-HOTrE). To parse this new data type, a custom-built R package (quantMSImageR) was developed with functionality to label regions of interest as well as quantify and analyze lipid distributions. The spatial distributions quantified by DESI-MRM were supported by LC-MS/MS analysis, with both indicating that 16-HOTrE and 12-HETE were associated with airways, while 12,13-DiHOME and arachidonic acid were mapped to parenchyma. This study realizes the potential of targeted MSI to routinely map low-abundance oxylipins with high specificity at scale.

## Introduction

Lipidomics has become an established technique to investigate disease pathways at the molecular level.^1^ Standard analytical approaches involve liquid chromatographic (LC) separation of homogenate samples, followed by mass spectrometry (MS) analysis to infer lipid concentration. While valuable, these approaches are unable to spatially map lipids to specific anatomical and cellular regions, instead providing data from the total tissue comprising cell and tissue types with different lipid metabolism rates and pathways. Mass spectrometry imaging (MSI) defines a group of analytical techniques that directly measure analytes from the sample surface, retaining their spatial localization. This information can be coupled with histology to infer the biosynthesis and localization sites of molecules. MSI data is routinely acquired with high-resolution MS instrumentation (HRMS); however, the resulting images are often dominated by a few lipid classes (e.g., phospholipids, glycerolipids, sphingolipids)^2^ owing largely to their high abundance and ionizability. While these experiments have generated important findings,^3,4^ MSI literature reporting on low-abundance lipids remains sparse.

Oxylipins are a broad class of bioactive lipids that are synthesized via oxygenation of fatty acids.^5^ They include a number of potent signaling molecules that regulate inflammation and exert pathophysiological function in multiple diseases.^6^ The most studied class of oxylipins is the eicosanoids (e.g., prostaglandins), which are formed from C-20 polyunsaturated fatty acids (PUFAs) (e.g., arachidonic acid). The analogous compounds formed from C18 fatty acids are termed octadecanoids and include linoleic and α-linolenic acid-derived compounds.^7^ Oxylipin dysregulation is closely associated with inflammatory diseases such as asthma.^8,9^ Inflammation is mediated by complex interactions between immune cells (e.g., mast cells, eosinophils) and structural cells (e.g., epithelial cells, airway smooth muscle cells) in a trigger and tissue-dependent manner that results in unique oxylipin profiles.^10^ There is a need to link specific cell types within a tissue to oxylipin formation in order to understand the physiological context, which is key to understanding these complex oxylipin signaling cascades.

In order to image low-abundance compounds, targeted or multiple-reaction-monitoring (MRM)-based MSI has recently been developed. This method uses desorption electrospray ionization (DESI), an ambient ionization method,^11^ coupled to a triple quadrupole mass spectrometer to perform DESI-MRM. This approach increases the sensitivity and specificity of the data acquisition. However, open-source tools for data analysis are focused on untargeted (HRMS) MSI data, generally importing data from the open-source MSI data format, imzML,^12^ which is not directly compatible with MRM-based MSI data. Accordingly, to realize the utility of DESI-MRM-based MSI, there is a need for software to perform advanced interrogations of spatial data.

**Figure 1:**
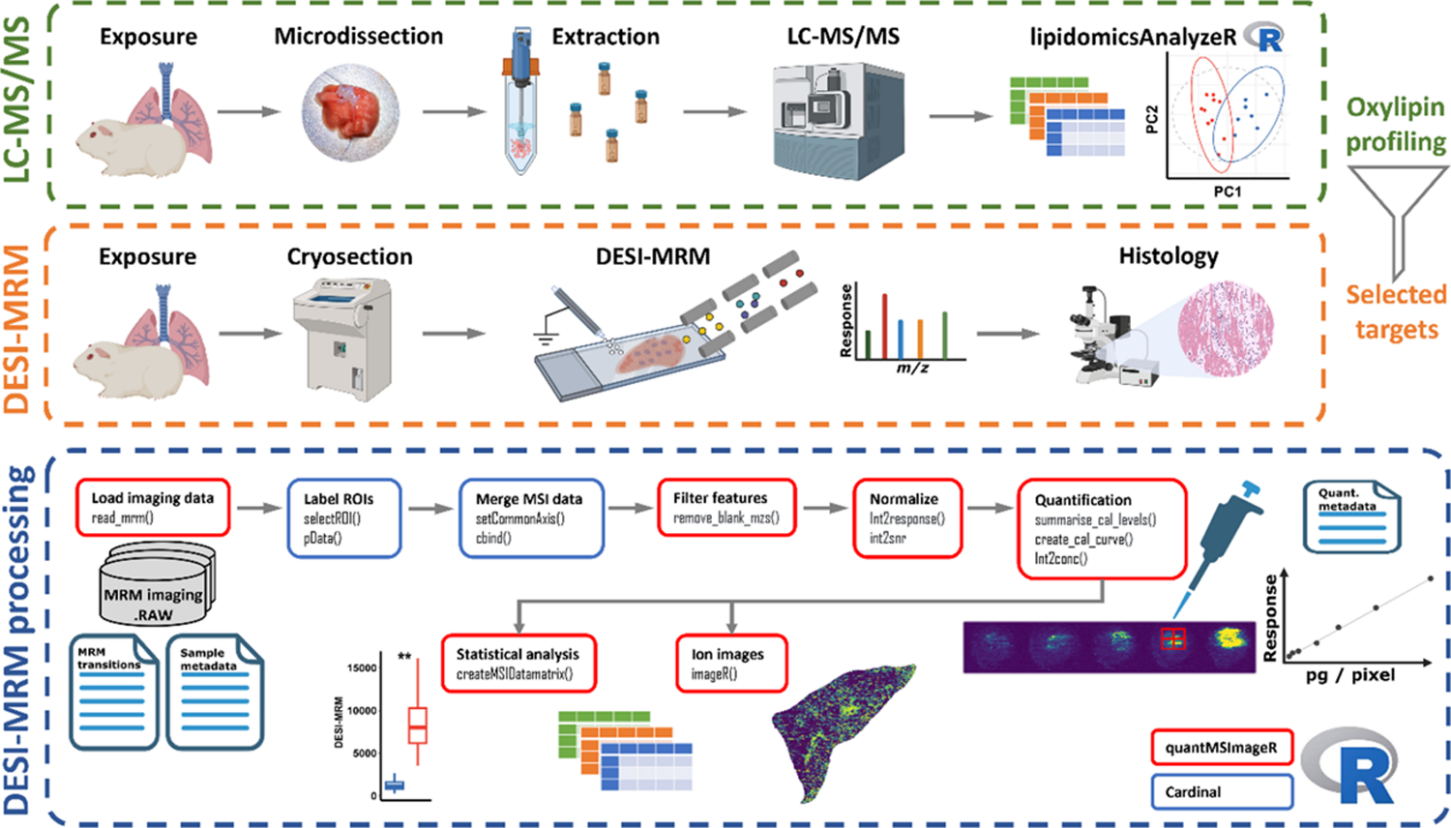
Complete workflow for spatially mapping oxylipins in pulmonary tissue. (1) The first step is to perform LC-MS/MS oxylipin profiling following microdissection of guinea pig lung tissue. The tissue can be from in vivo or ex vivo exposed animals or tissue, respectively. (2) In the second step, the LC-MS/MS data are used to select oxylipin targets to map at the surface of analogous tissue using DESI-MRM. The same cryosections can be stained for histology. The tissue can be from in vivo or ex vivo exposed animals or tissue, respectively. (3) Lastly, the DESI-MRM data and relevant meta files in regard to the samples, MRM transitions, and quantification are parsed to quantMSImageR to process, quantify, and analyze the data.

The aim of this study was to develop a targeted MSI workflow using DESI-MRM to spatially map oxylipins (Figure 1). Inflamed lung tissue was used to demonstrate the utility of the method in investigating oxylipin dysregulation. We first determined oxylipin heterogeneity in isolated lung tissue (airways and parenchyma) by LC-MS/MS, then spatially mapped selected lipids by DESI-MRM. An R package, quantMSImageR, was subsequently developed to process and analyze the novel imaging data type. This study presents the first development of a workflow to use DESI-MRM for routine mapping of low-abundance oxylipins.

## Experimental Section

### Chemicals and Reagents

For the *in vivo* and *ex vivo* guinea pig experiments: house dust mite (HDM) extract was purchased from Greer Laboratories (Lenoir, Lot#394845); Krebs-Henseleit buffer, compound 48/80, CaCl_2_, and NaHCO_3_ from Sigma-Aldrich (St. Louis); salbutamol hemisulfate from Tocris Bioscience (Bristol, U.K.); Dulbecco’s Modified Eagle’s Medium/Nutrient Mixture F-12 Ham, Penicillin-Streptomycin, and PBS from Gibco/Life Technologies Limited (Paisley, U.K.); Pentobarbital from Apotek Produktion & Laboratorier AB (Huddinge, Sweden). For LC-MS/MS and DESI-MRM data acquisition: water was used from an in-house Milli-Q system; Optima LC-MS/MS grade methanol (MeOH), acetonitrile (ACN), isopropanol (IPA) and acetic acid 99.5% were purchased from Fisher Chemical (Stockholm, Sweden); gelatin from porcine skin and carboxymethylcellulose sodium salt (CMC) were acquired from Merck (Darmstadt, Germany). For LC-MS/MS analyses, oxylipin standards were purchased from either Larodan (Solna, Sweden), Cayman Chemical (Ann Arbor, MI), or synthesized in-house as described by Quaranta et al.^13^ Oxylipin nomenclature is provided in Table S1.

### Guinea Pig Experiments

Female Dunkin–Hartley guinea pigs (Envigo, Horst, Netherlands) were accommodated in the animal facility at the Astrid Fagræus Laboratory, Karolinska Institutet, with *ad libitum* access to food and water. Diet was a combination of TEKLAD Global guinea pig feed (Inotiv/Envigo; Indiana), fresh fruit and vegetables, hay, and straw irradiated to prevent infections from wild rodents (Ssniff; Soest, Germany) and chew blocks (Tapvei; Harjumaa, Estonia). For the *in vivo* model, sensitization to house dust mite (HDM) was achieved through two intraperitoneal (i.p.) injections (50 μg each) and two intranasal (i.n.) instillations of HDM extract on day 1 (50 μg) and 4 (100 μg). Two weeks after sensitization, the animal was anesthetized using isoflurane inhalation and intranasally challenged with 25 μg of HDM dissolved in 200 μL of PBS twice a week for 2 consecutive weeks. Figure S1 depicts the HDM sensitization and challenge protocol.

Euthanization of naïve and *in vivo* HDM-exposed guinea pigs was performed with an overdose of pentobarbital (200 mg/kg). Upon cessation of vital signs, the lungs were perfused with ice-cold Krebs-Henseleit buffer containing the β_2_-adrenoceptor agonist salbutamol (1 μM). Subsequently, the heart-lung package was carefully removed. All experiments were approved by the Stockholm ethics committee (permit number: 10973-2019) and performed in accordance with local regulations.

### Preparation of *Ex Vivo* Challenged Guinea Pig Lung Tissue for LC-MS/MS

Lungs from three naïve guinea pigs (500–800 g, 180–200 days old) were microscopically dissected into airways and parenchyma and then submerged in ice-cold PBS. Tissue samples (20 mg) and blank wells excluding tissue were stimulated with 150 μg/mL of compound 48/80 (C48/80) in 1 mL of Dulbecco’s modified Eagle’s medium/nutrient mixture F-12 Ham supplemented with penicillin (100 IU/mL) and streptomycin (100 mg/mL) in a 48-well plate at 37 °C with a carbogen atmosphere (95% O_2_ and 5% CO_2_). After 1 h incubation, 250 μL of ice-cold MeOH/water (95:5 v/v) was added to 2 mL tubes with ZrO beads, to which the tissue samples were added. Samples were homogenized in a Fisherbrand Bead Mill 24 tissue homogenizer for 9 cycles of 15 s, with samples placed on dry ice between each cycle to limit temperature rise. 250 μL of ice-cold MeOH/water (95:5 v/v) was added to each sample, which was placed in an ultrasound bath with wet ice and sonicated for 15 min, then centrifuged at 12,000 *g* for 15 min at 8 °C. 90 μL of supernatant from the samples were added to LC-MS vials, with 10 μL of internal standard mixture (see below) and 10 μL of water. A further 20 μL of the supernatant was used to generate quality control samples (QCs). QC and extraction blank samples were prepared as for the tissue samples.

### LC-MS/MS Data Acquisition

LC-MS/MS profiling of oxylipins in tissue extracts was performed by two methods from reinjection of the same sample. Octadecanoids were measured with a modified version of the LC-MS/MS method published by Quaranta et al.^13^ Briefly, a Waters Acquity Premier BEH C18 with VanGuard FIT, 2.1 × 100 mm^2^, 1.7 μm column held at 40 °C with H_2_O + CH_3_COOH 0.1% v/v and ACN:IPA (90:10 v/v) for mobile phases A and B, respectively, was used for separation. Gradient elution was performed at 0.35 mL/min with 65% of A as the starting condition, linearly decreased to 58% between 1 and 2.5 min, to 50% at 4 min, to 35% at 7.5 min, to 25% at 11 min, and to 0% at 11.5 min. The column was then washed with 100% solvent B until 13.5 min and equilibrated to initial conditions at 13.6–15 min. The eluent was analyzed using a Waters Xevo TQ-XS (Waters, Milford, MA) with the addition of 7 detectable MRM transitions outlined in Table S2. The MS source was operated in negative-ion ESI mode under the following conditions: capillary voltage: 2.4 kV; source offset: 30.0 V; source temperature: 150 °C; desolvation temperature: 600 °C; cone gas flow: 150 L/h; desolvation gas flow: 1000 L/h; and nebulizer gas pressure: 7.0 bar. MS analyses were performed in negative MRM mode, with the collision energy, cone voltage, and dwell time manually optimized for each octadecanoid. One transition per analyte was selected based upon sensitivity and selectivity (Table S2).

Eicosanoids were measured by using the same instrumentation and mobile phases. However, the column was held at 35 °C, and the gradient elution was performed at 0.425 mL/min with 65% of A as the starting condition, linearly decreased to 60% at 2.1 min, to 58% at 3.5 min, to 50% at 5.5 min, to 27.5% at 11.5 min, and to 0% at 11.7 min. The column was then washed and re-equilibrated as for the octadecanoids. MRM transitions were as described by Kolmert et al.;^10^ however, polarity switching was used to detect cysteinyl leukotrienes (CysLTs) in positive ion mode and other eicosanoids in negative-ion mode from the same injection.

Internal standard mixtures were prepared as described previously for the octadecanoid^13^ and eicosanoid^10^ methods and mixed 1:1 to create a combined mixture (see Table S3 for final concentrations of internal standards). Standard curves were generated by injecting 10 and 11 calibration mixtures (for eicosanoids and octadecanoids, respectively) at the start and end of each acquisition. Preparation of the mixtures follows previously reported protocols. Briefly, the standard mixture was mixed with 10 μL of the combined internal standard mixture (to match the study samples) with a final solvent composition MeOH:water (84:16 v/v).

### LC-MS/MS Data Processing and Analysis

All peaks were integrated and quantified (pg/mg of tissue) from calibration curves using a TargetLynx (Waters, Milford, MA). An in-house R tool (https://github.com/targeted-lipidomics/lipidomicsAnalyzeR) was used to further process the data, comprising the following steps: (i) filter features based on signal:noise (S/N) > 5; (ii) filter compounds based upon presence in samples > 5-fold levels in blanks; (iii) evaluate technical variance based upon %RSD < 15% in QC samples; (iv) combine data from eicosanoid and octadecanoid acquisitions into a single data matrix; and (v) perform univariate and principal components analysis (PCA). For PCA, missing values were imputed as 20% of the minimum value for the given feature in the entire sample set and Pareto scaled. For univariate analysis, the Mann–Whitney–Wilcoxon test was performed with Benjamini and Hochberg False Discovery Rate (FDR) correction.

### Preparation of *In Vivo* Exposed Guinea Pig Lung Tissue for DESI-MRM

An individual female Dunkin–Hartley guinea pig (320 g, 30 days old) was euthanized 5 days after the last HDM challenge to collect lung tissue and liver tissue (for quantification purposes) for cryosectioning. The tissue was kept in PBS < 1 h post sacrifice before being transferred to 25 × 20 × 5 mm^3^ Tissue-Tek Cryomolds, embedded in preprepared 9% gelatin + 1% CMC media, and flash-frozen in liquid nitrogen. The embedding media was sonicated to remove gas and kept fluid using a hotplate stirrer at 35 °C under stirring to enable easy transfer to the molds with a Pasteur pipet and ensuring complete coverage of the tissue.^14^ The frozen tissue was equilibrated in an Epredia CryoStar NX70 cryostat at −15 °C, sectioned to 16 μm with an Epredia MX35 Ultra Microtome Blade and thaw mounted onto Corning single frosted 75 × 25 mm^2^ glass slides to be stored at −80 °C prior to DESI-MRM analysis and staining.

### DESI-MRM Data Acquisition

DESI-MRM data acquisition was performed with a Waters DESI-XS ionization source coupled to a Waters Xevo TQ Absolute (TqA) mass spectrometer. MeOH/water (95:5 v/v) + 0.1% v/v acetic acid was pumped from a Waters nanoAcquity Binary Solvent Manager at 2 μL/min, through a M-Class Symmetry C18 Trap Column (5 μm, 180 μm × 20 mm) to the DESI sprayer, where high voltage caused the solvent to electrospray. The electrosprayed solvent was pneumatically directed toward tissue sections by a flow of nitrogen gas at 75° to the *x*,*y*-sample stage. The sample was positioned on the DESI sample stage, where wetting of the analysis area (pixel) occurred from the impact of the charged solvent, followed by the desorption of analytes at the surface of the tissue and subsequent charge transfer. Droplets containing the desorbed analyte ions, termed secondary droplets, were then directed toward the heated transfer line (HTL) following the continued impact of the pneumatically directed solvent on the wetted region and were directed to the TqA for analysis.

DESI parameters were optimized to the octadecanoid 9-HODE (MRM transition in Table S4) in guinea pig lung tissue, which was selected due to its high abundance. Geometric parameters, comprising the DESI sprayer to sample distance and source-to-sample angle, were adjusted to ensure that the trajectory of secondary DESI droplets was directed toward the HTL, ensuring time for ionization of the lipids while minimizing the increase in analysis region caused by free jet expansion. Additionally, the HTL temperature, voltage at the DESI sprayer, and flow of nitrogen gas were optimized and stored in a Waters .ipr file, with adequate performance at 200 °C, 0.67 kV, and 0.15 MPa, respectively, in negative ionization mode. Prior to analysis of biological samples, movement of the *x*,*y*-plate and correct positioning of the sample stage for the optimized DESI-MRM setup was assessed by analyzing black ink (*m*/*z* 666.06).

DESI-MRM experiments were set up using HDImaging v1.7 and DESI Method Editor (Waters) as follows:^15^ (i) cryosections were thawed under nitrogen for >10 min to limit oxidation; (ii) the analysis region was marked on the underside of the tissue with a marker pen as well as the edges of the slide as fiducial markers; (iii) optical images of the tissue sections were generated, imported and registered in DESI Method Editor using the fiducial markers; (iv) the analysis region was selected; (v) MRM transitions of selected oxylipins taken from the LC-MS/MS method (Table S4) were defined by their precursor *m*/*z*, product *m*/*z* collision energy (eV), and cone voltage; and (vi) pixel size (50 μm^2^) and scan rate (4 Hz) for the experiments were input as shown in Figure S2. DESI Method Editor calculated the dwell times automatically, resulting in 38 ms/pixel for each MRM transition. The experiments were then imported into MassLynx v4.2 (Waters), and the optimized parameters stored in the .ipr file were used to acquire the DESI-MRM data.

### Histological Staining

After DESI-MRM analysis, the same tissue sections were used for hematoxylin and eosin or May–Grünwald Giemsa staining to visualize tissue types (airways, vessels, and parenchyma) or cell types (eosinophils), respectively. The stained slides were scanned using a Zeiss AxioScan Z1 (Biomedicum Imaging Core) at a 20× magnification.

### DESI-MRM Data Processing and Analysis

The data were processed within MassLynx, creating a .txt file containing MS and spatial information. This file was then processed using quantMSImageR (https://github.com/targeted-lipidomics/quantMSImageR), a software package developed to process DESI-MRM data that is based on Cardinal, an established software for processing untargeted MSI data.^16^ Data processing used tools from both quantMSImageR and Cardinal (depicted in Figure 1): (i) creating an ‘MSImagingExperiment’ object containing pixel, lipid, and sample metadata from the MassLynx .txt file; (ii) setting a common MRM axis for all MSI data sets in the study to merge downstream; (iii) selecting and labeling regions of interest (ROIs) and background pixels (pixels from the same DESI-MRM acquisition excluding tissue, i.e., embedding media) in each tissue section; (iv) combining imaging experiments in the study; (v) removing blank MRM channels; (vi) calculating the S/N of MRMs in each pixel and filter (S/N > 3 for detection and >5 for quantification), where noise was defined as the mean response of the background pixels; (vii) generating ion images for each MRM transition, where the response, S/N, or concentration at each pixel is a scaled color from the vidris color map; and (viii) outputting conventional omics data matrices of MS response values, with lipids in rows and either pixel or ROI in columns, along with corresponding lipid and pixel metadata for compatibility with existing omics tools for statistical analyses. Specifically in this work, data analysis comprised the visualization of lipid distribution and univariate analyses. For univariate analyses, spectra from 50 pixels associated with each tissue type (airways and parenchyma) were extracted, and the lipid response was visualized as boxplots with associated *P*-values determined via a Mann–Whitney–Wilcoxon test.

### Quantification of 12,13-DiHOME by DESI-MRM

Glass slides containing sequential liver sections were thawed, and eight spots were marked on the underside of the glass slides. Eight solutions of 12,13-DiHOME (0, 0.5, 1, 5, 10, 25, 150, and 400 ng/mL) were prepared in 50:50 MeOH:water and 1 μL of each were spiked onto the premarked positions. This resulted in 0, 0.5, 1, 5, 10, 25, 150, and 400 pg of 12,13-DiHOME at each spot, respectively. The sample was then analyzed by DESI-MRM with optimized parameters similar to those for the experimental samples. The data was then parsed into quantMSImageR as described above, with the addition of a .csv file indicating the amount of compound at each calibration level, and processed as follows: (i) ROIs were selected for each calibration spot and labeled; (ii) for each calibration ROI the number of pixels and mean MS response were calculated; (iii) a linear model was generated for the MS response vs concentration (pg/pixel); and (iv) the concentration of 12,13-DiHOME in lung tissue was estimated from the linear model using the MS response at each pixel sequentially.

## Results and Discussion

### Oxylipin LC-MS/MS Profiling

**Figure 2:**
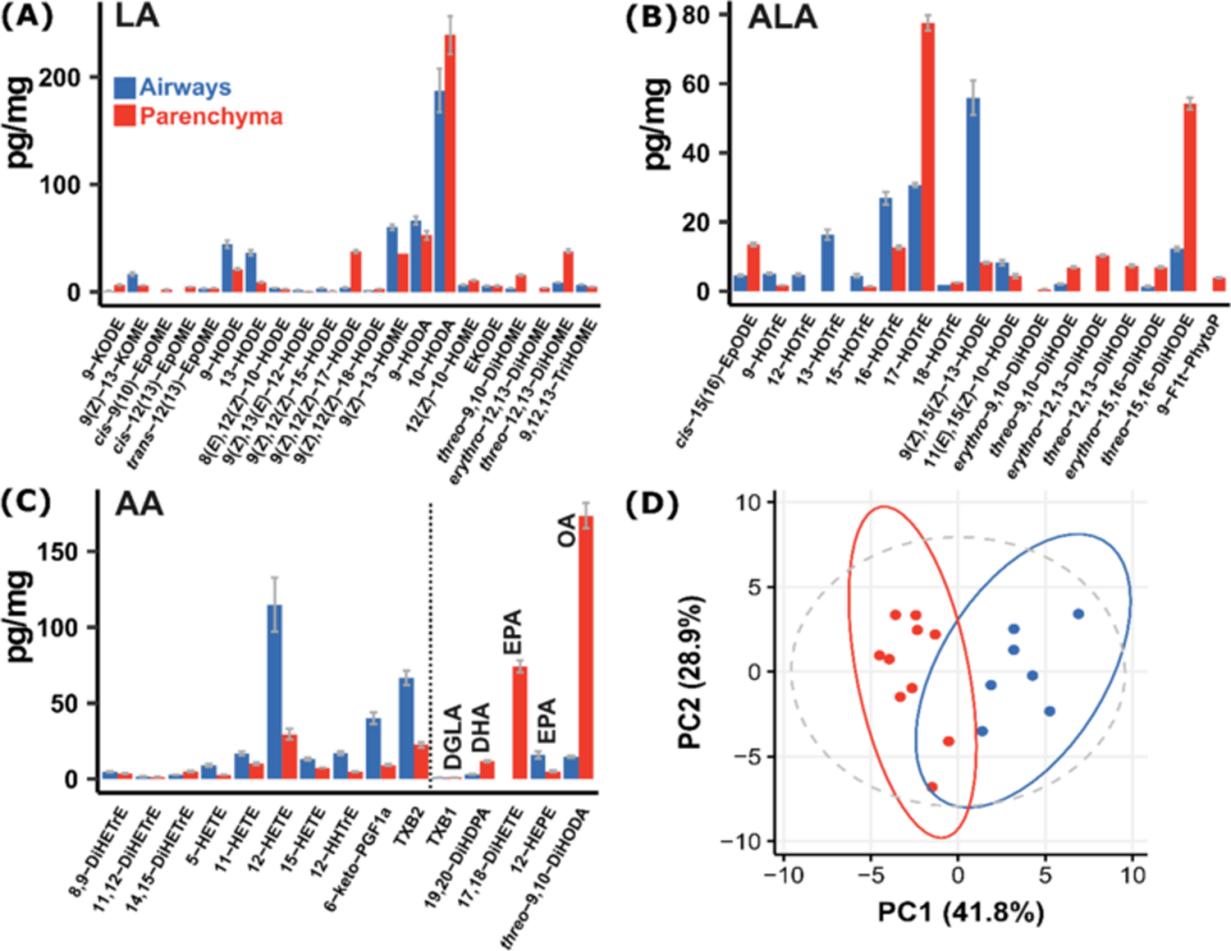
LC-MS/MS analysis of oxylipin extracts from isolated guinea pig lung tissue. (A) Oxylipins derived from linoleic acid (LA); (B) oxylipins derived from α-linolenic acid (ALA); and (C) oxylipins derived from arachidonic acid (AA), dihomo-γ-linolenic acid (DGLA), docosahexaenoic acid (DHA), eicosapentaenoic acid (EPA), and oleic acid (OA). (D) Principal component analysis (PCA) scores plot of all 53 quantified oxylipins. Airways (blue) and parenchyma (red). *N* = 3 biological replicates, *n* = 4 technical replicates.

**Figure 3:**
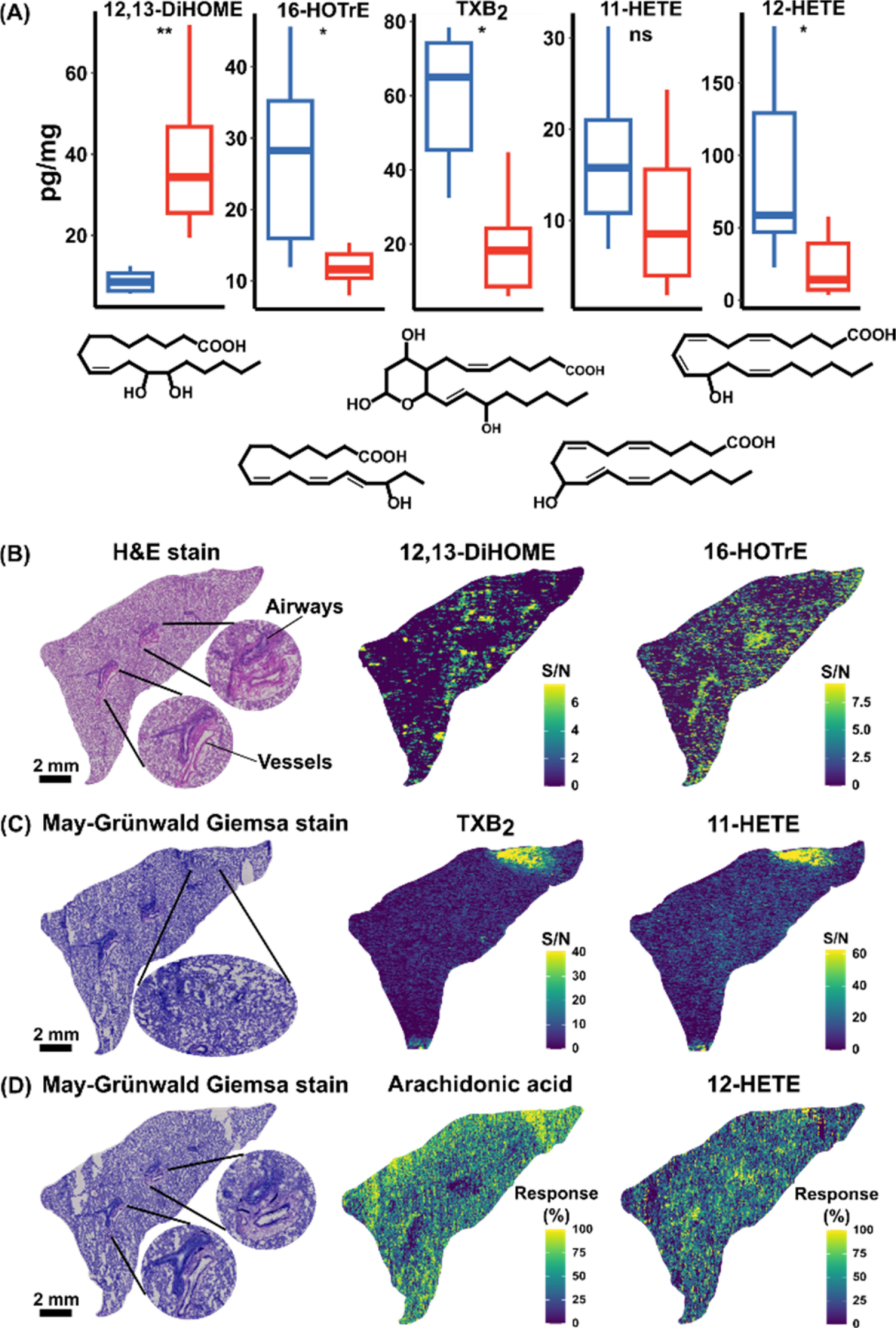
DESI-MRM imaging of selected oxylipins in guinea pig lung tissue. (A) LC-MS/MS analysis of 5 oxylipins (labeled above each boxplot with the corresponding structure provided below) in extracts from ex vivo C48/80-exposed guinea pig lung parenchyma (red) and airways (blue). Significance was determined using a Benjamini and Hochberg false discovery rate (FDR)-corrected Mann–Whitney–Wilcoxon test (ns = not significant, *P < 0.05, **P < 0.01). (B) H&E stained cryosection showing parenchyma, airways, and vessels in guinea pig lung tissue and the associated ion images from the linoleic acid-derived soluble epoxide hydrolase (sEH) product 12,13-DiHOME and α-linolenic acid-derived autoxidation product 16-HOTrE. (C) May–Grünwald Giemsa stained cryosection showing parenchyma, airways, and vessels in guinea pig lung tissue and the associated ion images from the cyclooxygenase (COX)-derived TXB_2_ and 11-HETE. (D) May–Grünwald Giemsa stained cryosection showing parenchyma, airways, and vessels in guinea pig lung tissue and the associated ion images from arachidonic acid and its 12-lipoxygenase (12-LOX) product 12-HETE.

The first step in the workflow (Figure 1) was to determine the oxylipin content of the tissue by quantifying the eicosanoid and octadecanoid profiles using LC-MS/MS. The results were then used to select the oxylipins for DESI-MRM imaging. To ensure a robust oxylipin signature, guinea pig tissue was exposed to C48/80, a Mas-related G protein-coupled receptor member X2 (MRGPRX2) agonist, known to stimulate oxylipin formation and induce smooth muscle contraction through localized mast cell activation.^17^ The LC-MS/MS analysis of oxylipins generally includes a concentration step due to their low levels; however, this procedure was not performed because the aim was to determine which oxylipins could be detectable by DESI-MRM (which inherently excludes concentration steps). A total of 13 eicosanoids and 39 octadecanoids were detected > LLOD in either guinea pig parenchyma or airways (Figure 2A–C). PCA was performed on these combined data, showing that the oxylipin profile was distinct between these tissue types (Figure 2B, loadings Figure S3). Based upon concentration and fold change in each tissue type (Table S5), 5 oxylipins were selected for DESI-MRM analysis: 12,13-DiHOME, 16-HOTrE, 11-HETE, 12-HETE, and TXB_2_ (see Figure 3 for the chemical structures). This panel of oxylipins ranged in tissue concentration from 23–130 pg/mg and represents multiple PUFA pathways. The fold change (FC, airways:parenchyma) and FDR-corrected *P*-value were determined for each compound. The 12,13-DiHOME (12,13-dihydroxy-9*Z*-octadecenoic acid, FC = 0.23, *P* = 0.0067) is the combined cytochrome P450 and soluble epoxide hydrolase (sEH) product of linoleic acid. The 16-HOTrE (16-hydroxy-9*Z*,11*E*,14*Z*-octadecatrienoic acid, FC = 2.1, *P* = 0.020) has not been previously reported but is most likely an autoxidation product of α-linolenic acid. The 11-HETE (11-hydroxy-5*Z*,8*Z*,12*E*,14*Z*-eicosatetraenoic acid, FC = 1.7, *P* = 0.16) and 12-HETE (12-hydroxy-5*Z*,8*Z*,10*E*,14*Z*-eicosatetraenoic acid, FC = 3.9, *P* = 0.044) are formed from arachidonic acid, likely by cyclooxygenase (COX) and 12-lipoxygenase (12-LOX) activity, respectively. However, without data on stereochemistry, it is not possible to state the synthetic source of these compounds unequivocally. Lastly, TXB_2_ (thromboxane B_2_, FC = 3.0, *P* = 0.020) is synthesized via COX and subsequent thromboxane synthase activity.

### DESI-MRM Workflow

For the first time, we were able to image oxylipins in lung tissue. Previous efforts have imaged prostaglandins in mouse uterus using HRMS; however, silver nitrate additives were required to increase the sensitivity (∼30-fold).^18^ Targeted MSI offers routine detection of these low-abundance lipids at high scan speeds due to reduced chemical noise and dwell time of MRM transitions compared to HRMS. The use of a nanoBSM for the DESI solvent enabled stable spray for multiday data acquisitions, with addition of the M-Class Symmetry C18 Trap Column important for maintaining back pressure (>400 psi at 2 μL/min flow rate), which prevented image artifacts (Figure S4).

The improved S/N of MRM- compared to HRMS-based imaging methods enables lower abundant lipids to be routinely imaged, increasing the practical coverage of MSI. However, the increase in sensitivity is at the expense of the number of analytes that can be detected in a single scan with a maximum of 32 MRM transitions. This DESI-MRM workflow builds upon LC-MS/MS profiling. Mapping selected contextually important oxylipins with high sensitivity and acquisition speed enables more reliable biological inference due to a more reproducible DESI-MRM signal and additional biological replicates. The trade-off between speed and number of MRM transitions becomes even more important with increasing spatial resolution because acquisition times are a function of the squared pixel size and sensitivity related to the analysis area (i.e., analytes capable of being desorbed). DESI-MRM at the single-cell level necessitates focusing acquisition on small tissue areas, ideally defined by a prior survey scan at lower spatial resolution, which is feasible because DESI is inherently nondestructive. In this work, we reach a modest 50 μm^2^ pixel size for up to six MRM transitions, which optimizes the duty cycle for the acquisition speed and sensitivity. However, increased spatial resolution to <10 μm^2^ pixel size has been achieved with DESI (Emrys Jones, personal communication), primarily dependent on providing DESI solvent at a stable low flow (500 nL/min).

DESI-MRM offers higher specificity than untargeted approaches, with the latter often relying on annotations from high-resolution *m*/*z* measurements only (putatively characterized compound class). However, a single MRM transition is sometimes insufficient to distinguish the isomers. This is particularly problematic for oxylipins, for which there are numerous isomers that can be formed via multiple pathways. For example, Weigand et al. reported that they discriminated the arachidonic acid-derived oxylipins 9-HETE from 11-HETE in rat kidney using a custom-designed nano-DESI-MRM employing a single transition for each lipid (transitions in Table S4).^19^ However, previously published work for the HETE series shows that the MRM transition (319.2 > 179.1) is common to both 9-HETE and 12-HETE, and either a qualifier ion or orthogonal analytical approach (e.g., chromatography) is required to distinguish these isomers.^10^ Similar potential confounders are possible with many oxylipins exhibiting the same MRM transitions. For example, the transition 319.2 > 167.1 has been reported for both 9-HETE and 11-HETE.^20^ A common strategy to distinguish isomers is the use of multiple transitions. We attempted this approach for 12,13-DiHOME by mapping both the 313.2 > 183.1 and 313.2 > 129.2 transitions on liver tissue spiked with the standard (Figure S5). While yielding a Pearson’s correlation of 0.97, this approach was unsuccessful for detecting endogenous oxylipins due to their low concentration (Figure 3B–D). In some pixels, only a single transition of the endogenous oxylipin was detected, preventing spatial localization. However, by coupling the DESI-MRM to orthogonal LC-MS/MS analysis of analogous tissue, our workflow was able to determine that 9-HETE levels were 114 ± 18 and 29 ± 4 pg/mg in the airways and parenchyma, respectively (Table S5).

To parse, process, and analyze the DESI-MRM data, we developed an R package (quantMSImageR) that is based upon the Cardinal package.^16^ The software includes functionality to: read MRM-based MSI data; filter MRM transitions < LLOD; calculate S/N of MRM transitions based on background pixels and filter based on this; estimate concentration within tissue based on replicated calibration curves; and output data matrices of lipid response or concentration at each pixel or ROI as described in the methods. Ion images are generated with flexible options for scaling pixel intensities to enable visualization of lower abundant pixels, such as quantile suppression as well as quantile thresholding to remove background pixels. Additionally, functionality to normalize to internal standards is included, which could benefit the targeted MSI community; however, this function was not utilized in the current study. The example computational workflow for quantMSImageR is available from GitHub as a vignette and shows the commands required to perform each step outlined in this study and output figures and tables.

**Figure 4:**
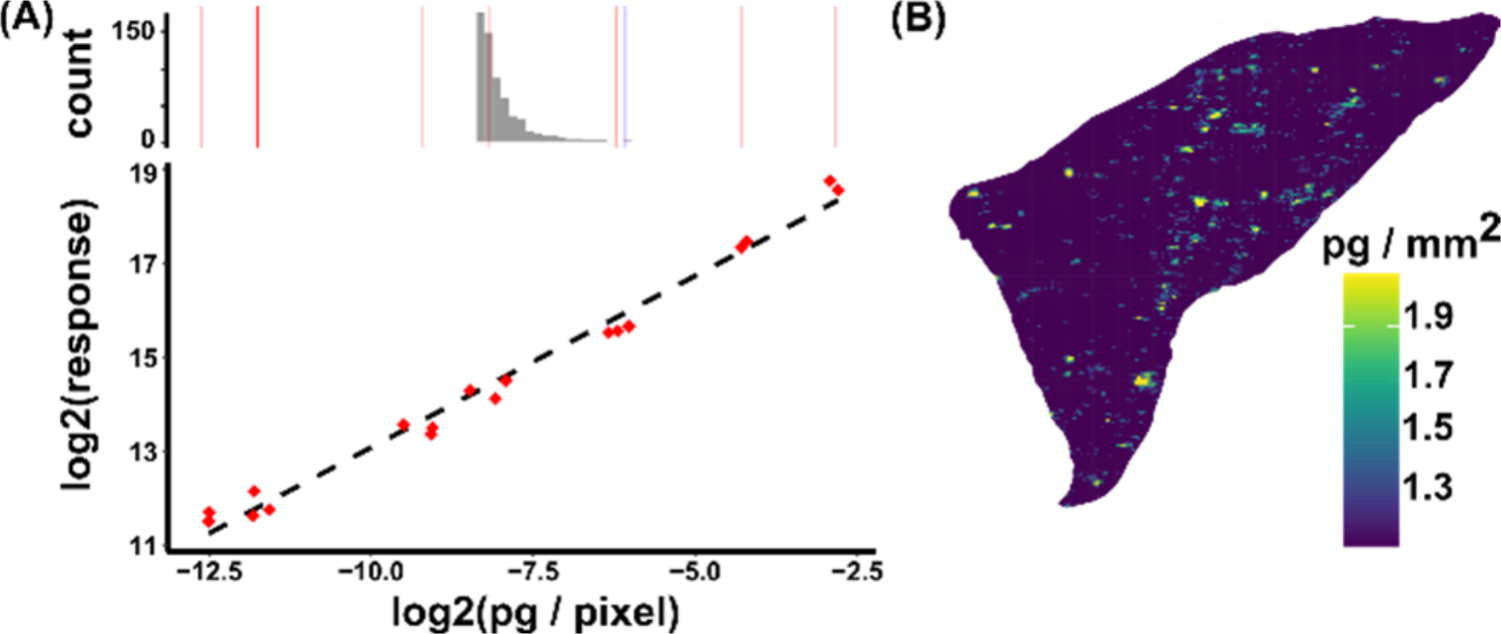
Estimated quantification of 12,13-DiHOME in guinea pig lung tissue by DESI-MRM. (A) Mass spectrometry response vs concentration (pg/pixel) on a logarithmic scale (base 2) for 12,13-DiHOME calibration standards spotted onto guinea pig liver (0, 0.5, 1, 5, 10, 25, 150, 400 pg). A histogram is shown on the same *x*-axis displaying the concentration of tissue pixels (maximum pixel value indicated with a blue vertical line) above S/N > 5. The average pg/pixel of the triplicate calibration levels are depicted with red vertical lines. (B) Ion image of the octadecanoid 12,13-DiHOME in guinea pig lung tissue showing concentration as pg/pixel for all pixels with S/N > 5.

The analytical workflow includes estimated quantification measured in pg/pixel. This approach provides an estimation of the oxylipin concentration at the tissue surface, which we demonstrate with 12,13-DiHOME. Lung tissue is heterogeneous, rendering it challenging to spot a calibration curve across a tissue section with a similar composition. Accordingly, the DESI-MRM analysis of triplicate standard curves was prepared on liver sections to act as a pseudo-biological matrix to mimic the physical and chemical properties of the lung, accounting for extraction and ion suppression effects. The curves yielded an average linear response with *R*^2^ = 0.97 from 0.2 to 400 pg spots at 0.143–155 fg/pixel (Figure 4A and Table S6). This enabled quantification of all pixels containing 12,13-DiHOME in terms of pg/pixel with S/*N* > 5 in guinea pig lung tissue (Figure 4B). The S/N threshold was increased from 3 to 5 for the purposes of quantification, according to FDA bioanalytical guidelines.

The histogram in Figure 4A shows the estimated 12,13-DiHOME concentration distribution of tissue pixels. Replicate calibration spots have different theoretical concentrations because the concentration is relative to the number of pixels within the calibration spot, and spot sizes were not uniform and instead distributed over a variable number of pixels. To limit the variability, 50% water was added to the standard (MeOH) to increase the surface tension and thus reduce the wettability. This variability could be further reduced with the use of dedicated automated spotters^21^ and minimize the subjectivity of pixel selection to improve quantification. An additional challenge for quantification (and general data quality) with MSI is the heterogeneous nature of sample surfaces, which causes distinct matrix effects as well as desorption/extraction artifacts in DESI-MRM data. The heterogeneity cannot be fully corrected by internal standards due to the inability to homogeneously distribute the compounds through tissue sections. Källback et al. reported a quantitative MALDI-MSI approach for drug metabolites in mouse brain, which was cross-validated against LC-MS/MS.^21^ To achieve quantitative MSI, standards were robotically spotted in 87 pL droplets with high accuracy to enable the generation of calibration curves from specific subregions of tissue for matrix matching, with post spraying of internal standards to account for technical variance and degradation of analytes and to mimic the surface desorption. However, this approach requires an additional time-intensive step to spray the internal standard at the surface (a prerequisite of MALDI, but not DESI) and does not fully match the surface interactions or degradation pathways of the analytes. We postulated that a more compatible approach for DESI would be to spike a technical internal standard into the DESI solvent to correct for instrument variance and ion suppression. For this purpose, we used the compound CUDA (12-[[(cyclohexylamino)carbonyl]amino]-dodecanoic acid), which is a stable and inexpensive chemical that ionizes well in both positive and negative modes. To assess this approach, we mapped the intensity of CUDA (5 ng/mL in the DESI solvent) across blank slides with highly ionizable black ink (a compound used for system suitability assessment that is compatible with DESI^15^) deposited in four distinct regions. We observed that the relative intensity of CUDA was reduced in regions where the ink was deposited (Figure S6). However, while CUDA could correct for ion suppression and technical variance, our initial efforts to implement a normalization strategy in lung tissue by dividing each analyte intensity by CUDA intensity on a pixel basis were unsuccessful. The normalization corrected global trends, but caused artifacts localized to the airways due to the absence of tissue (Figure S7). Tissue heterogeneity-related matrix effects were also observed by Taylor et al., who compared the intensity of homogeneously distributed olanzapine in mouse brain by MALDI and DESI. They reported matrix effects across cerebellum, hippocampus, and white matter, but concluded that DESI causes significantly less ion suppression relative to MALDI.^22^ Based upon these findings, and to avoid local artifacts around airways, we opted to omit the use of technical internal standard normalization in this work; however, further effort should be placed on developing a computational strategy for implementation.

### DESI-MRM Comparison to LC-MS/MS

The *in vivo* HDM guinea pig model is known to activate mast cells and cause smooth muscle contraction,^23^ thus serving as a useful case for the DESI-MRM mapping of oxylipins. The relative distributions of the S/N of octadecanoids 16-HOTrE and 12,13-DiHOME at the surface of the lung tissue are visualized in Figures 3B and S8. The 16-HOTrE was visually associated with the airways, while 12,13-DiHOME was more concentrated in the parenchyma (*P* = 2.2 × 10^–8^). These tissue-specific oxylipin distributions from DESI-MRM match the corresponding LC-MS/MS analyses of isolated tissue (Figure 3A). The use of LC-MS/MS also enabled *threo- and erythro*-12,13-DiHOME (Figure S9) to be distinguished, with the *threo-*stereoisomer >10-fold more concentrated than the *erythro*-stereoisomer in the parenchyma, and the *erythro*-12,13-DiHOME was < LLOD in the airways. Data for both stereoisomers are combined in Figure 3A, with the *threo-stereo*isomer contributing to ∼90% of the total quantity of 12,13-DiHOME detected in parenchyma and 100% of that detected in the airways.

The data demonstrate the ability of MSI to detect intratissue oxylipin heterogeneity, which could not be observed by LC-MS/MS of isolated tissue. Specifically, for the COX-derived oxylipins, TXB_2_ was concentrated in the airways, while 11-HETE was homogeneous throughout the lung section based upon the LC-MS/MS analysis. However, DESI-MRM showed that the distributions of both oxylipins were concentrated to a specific region of the parenchyma (Figure 3C). This highlights the major limitation of analyzing isolated regions of tissue by LC-MS/MS and emphasizes the need to analyze tissue cross sections to better understand the oxylipin signaling cascades. The colocalization of TXB_2_ and 11-HETE is not surprising given that they are both COX products of arachidonic acid; however, the concentration within a specific region of lobe was interesting. Post DESI-MRM, we stained the tissue section with May–Grünwald Giemsa stain to screen for specific cell types in the region that could be responsible for this metabolism (e.g., thrombocytes, or inflammatory cells such as eosinophils). Based upon a lack of observed eosinophils, localization was likely caused by vessel rupture in the tissue and the ensuing platelet activation, which is reflected in the increased levels of COX products.

In Figure 3D, the ion images show arachidonic acid associated with parenchyma with no presence around the airways, while the putative 12-LOX product (12-HETE) is weakly associated within the airways. These findings were supported by the LC-MS/MS data, which showed an increase in 12-HETE levels in the airways compared to the parenchyma (*P* = 0.044). Note that the ion images show the DESI-MRM response (rather than S/N) due to the presence of arachidonic acid in the embedding media and thus background pixels. Additionally, the LC-MS/MS method used did not include arachidonic acid. Most arachidonic acid in mammals is esterified in phospholipids; however, it is noteworthy that the free nonesterified arachidonic acid is only associated with the parenchyma, while the pro-inflammatory 12-HETE was weakly associated with the airways in the inflamed lung.

A major limitation with the analysis of monohydroxy-containing oxylipins (e.g., HODEs, HETEs) is that the observed lipids may form via PUFA autoxidation, resulting in incorrect association with enzymatic biosynthesis. In this work, we concluded that the HETEs were enzymatically produced due to their differential distribution relative to arachidonic acid. If the primary route of formation was autoxidation, these oxylipins and arachidonic acid would be expected to have similar distributions in the lung. As an additional component to the workflow to provide conclusive evidence of enzymatic formation, the LC-MS/MS could be performed with chiral analysis.

To demonstrate the reproducibility of our workflow, we mapped oxylipins by DESI-MRM in three sequential sections (to act as pseudo-technical replicates) 6 months apart and performed duplicate analyses of the same section over 2 days. We show that 16-HOTrE was consistently associated with airways (Figure S10), TXB_2_ was localized to a specific vessel rupture in the parenchyma (Figure S11), and results were consistent 6 months apart.

### Limitations

Several challenges remain in realizing the full potential of targeted MSI, many of which also exist in the untargeted MSI community. Specifically: (i) the inability to resolve isomers given the incompatibility with chromatographic separation; (ii) the lack of robust quality control strategies because preparation of homogeneous study-specific tissue samples is not routinely feasible; (iii) quantification is only estimated because internal standards cannot be homogenously distributed within tissue to match the ionization and extraction of analytes; (iv) acquisition times are long compared to LC-MS/MS, which limits replication and renders the data subject to greater technical variability; and (v) single-cell spatial resolution is not yet routinely possible with DESI. Solutions have been proposed for these problems, such as: (i) employing ion mobility separation of analytes to resolve isomers;^24^ (ii) using ‘1546a Meat Homogenate’ as a standard reference material from NIST for quality control; and (iii) distributing an internal standard within the DESI solvent or across tissue sections, which could account for ion suppression and technical variance to improve quantification and reproducibility.^21^ However, these approaches have not yet been universally accepted by the MSI community and do not fully address the problems. Other issues require technological improvements, for instance, the initial work toward ‘Fast Mass Microscopy’ by Körber et al.,^25^ which can perform MSI at rates 2500 times faster than microprobe-mode (DESI and MALDI), albeit with a custom design that is not compatible with MRM detection mode.

There are limitations unique to this MRM-based MSI workflow. There is a low number of parallel transitions per acquisition to maximize sensitivity, and the lack of cell identification from histology hinders the biological inference of the MSI data. It is also important to note that the prostaglandins and leukotrienes were not detected by either LC-MS/MS or DESI-MRM. This is likely due to a combination of low endogenous levels and omission of a concentration step of the tissue extracts (as is normally performed for LC-MS/MS-based oxylipin quantification). In *ex vivo* studies with human lung tissue in which potent stimulations were performed with anti-IgE, DESI-MRM was able to detect prostanoids (data not shown).

## Conclusions

This study presents the first complete workflow for spatially mapping low-abundance oxylipins directly from tissue sections using commercially available instrumentation. The study includes optimized DESI parameters, MRM transitions, and development of a software tool for this data type. The minimal sample preparation required for DESI, increased sensitivity, and short dwell times for MRM transitions coupled with reduction in instrumentation costs compared with HRMS, opens the door for larger MSI studies to be conducted on a wider range of lipids, increasing both statistical power and effective lipidome coverage possible with MSI. Development of the quantMSImageR software was an important step in this workflow for processing oxylipin MSI data but is also applicable to the general community. A key point of the workflow is the use of orthogonal LC-MS/MS analyses, which were necessary to infer oxylipin detectability and distinguish isomers, ensuring selectivity of the MSI data. DESI-MRM analysis of oxylipins in the guinea pig lung tissue demonstrated that the workflow has sufficient sensitivity to spatially map these low-abundance oxylipins for the first time in lung tissue. This is an important step toward addressing a knowledge gap regarding the regulation of inflammatory processes in the airways via oxylipin signaling cascades.

## Data Availability

The *RAW DESI-MRM data are available from zenodo (https://zenodo.org/doi/10.5281/zenodo.10807653).

## Supporting Information Available

Description of oxylipin nomenclature; list of oxylipins in the LC-MS/MS method and their transitions; internal standard mixture composition for LC-MS/MS; list of DESI-MRM transitions; results from LC-MS/MS profiling; results from DESI-MRM quantification; details about *in vivo* guinea pig challenge; details about DESI-MRM experimental setup; PCA loadings from LC-MS/MS profiling; ion image artifact; colocalization of 12,13-DiHOME transitions; ion images depicting ion suppression; normalization of ion images with a technical internal standard; boxplots from DESI-MRM data; boxplots of 12,13-DiHOME stereoisomers from LC-MS/MS; ion images and H&E stains of sequential sections; and duplicate analyses. (PDF)
