# Supplementary material for: Development of a Desorption Electrospray Ionization–Multiple-Reaction-Monitoring Mass Spectrometry (DESI-MRM) Workflow for Spatially Mapping Oxylipins in Pulmonary Tissue
Source: Anal Chem. 2024 Oct 26;96(45):17950–9. doi: 10.1021/acs.analchem.4c02350 (PMC11561881; doi:10.1021/acs.analchem.4c02350)
Supplement: Supplementary file 1 — ac4c02350_si_001.pdf [file ac4c02350_si_001.pdf]

Supporting Information

Development of a desorption electrospray ionization – multiple-reaction-monitoring mass spectrometry (DESI-MRM) workflow for spatially mapping oxylipins in pulmonary tissue

Matthew. J. Smith[1,2], Mu Nie[3], Mikael Adner[3], Jesper Säfholm[1,2], Craig E. Wheelock[1,2,*]

[1]Unit of Integrative Metabolomics, Institute of Environmental Medicine, Karolinska Institutet, Stockholm, Sweden
[2]Department of Respiratory Medicine and Allergy, Karolinska University Hospital, Stockholm, Sweden
[3]Experimental Asthma and Allergy Research Unit, Institute of Environmental Medicine, Karolinska Institutet, Stockholm, Sweden

Table S1 – Nomenclature for oxylipins detected by LC-MS/MS S2
Table S2 – LC-MS/MS MRM transitions of oxylipins S4
Table S3 – Internal standards and their final concentration in the LC-MS/MS internal standard mixture S6
Table S4 – DESI-MRM transitions S8
Table S5 – LC-MS/MS data S9
Table S6 – Calibration curve preparation of 12,13-DiHOME for quantification S11

Figure S1 – *In vivo* guinea pig model S12
Figure S2 – DESI-MRM experiment set-up S13
Figure S3 – LC-MS/MS analysis of oxylipin extracts from isolated guinea pig lung tissue S14
Figure S4 – DESI-MRM imaging of SM(d18:1/16:0) in guinea pig lung tissue S15
Figure S5 – DESI-MRM imaging of two transition relating to the 12,13-DiHOME standard spotted on guinea pig liver sections S16
Figure S6 – DESI-MRM imaging of the technical internal standard and black ink across a blank slide S17
Figure S7 – DESI-MRM imaging of hexose in guinea pig lung tissue before and after correction to the technical internal standard S18
Figure S8 – DESI-MRM imaging of selected oxylipins in guinea pig lung tissue S19
Figure S9 – LC-MS/MS analysis of oxylipin extracts from isolated guinea pig lung tissue S20
Figure S10 – DESI-MRM imaging of 16-HOTrE across sequential sections and repeat acquisitions S21
Figure S11 – DESI-MRM imaging of TXB_2 across sequential sections and repeat acquisitions S22

***Corresponding author**
Craig E. Wheelock
Unit of Integrative Metabolomics
Institute of Environmental Medicine
Karolinska Institutet
SE-171 77 Stockholm, Sweden
Email: craig.wheelock@ki.se

Table S1 – Nomenclature for oxylipins detected by LC-MS/MS

Abbreviation	Compound name	Parent PUFA
8,9-DiHETrE	8,9-dihydroxy-eicosatrienoic acid	Arachidonic acid
11,12-DiHETrE	11,12-dihydroxy-eicosatrienoic acid	Arachidonic acid
14,15-DiHETrE	14,15-dihydroxy-eicosatrienoic acid	Arachidonic acid
5-HETE	5-hydroxy-eicosatetraenoic acid	Arachidonic acid
11-HETE	11-hydroxy-eicosatetraenoic acid	Arachidonic acid
12-HETE	12-hydroxy-eicosatetraenoic acid	Arachidonic acid
15-HETE	15-hydroxy-eicosatetraenoic acid	Arachidonic acid
12-HHTrE	12-hydroxy-heptadecatrienoic acid	Arachidonic acid
6-keto-PGF α	6-keto-prostaglandin F1α	Arachidonic acid
TXB_1	Thromboxane B1	Arachidonic acid
TXB_2	Thromboxane B2	Arachidonic acid
19,20-DiHDPA	19,20-dihydroxy-docosapentaenoic acid	Docosahexaenoic acid
17,18-DiHETE	17,18-dihydroxy-eicosatetraenoic acid	Eicosapentaenoic acid
12-HEPE	12-hydroxy-eicosapentaenoic acid	Eicosapentaenoic acid
9-KODE	9-oxo-octadecadienoic acid	Linoleic acid
9*(Z)*-13-KOME	13-oxo-9*(Z)*-octadecenoic acid	Linoleic acid
cis-12(13)-EpOME	*cis*-12(13)-epoxy-octadecenoic acid	Linoleic acid
EKODE	9-oxo-*trans*-12(13)-epoxy-octadecenoic acid	Linoleic acid
trans-12(13)-EpOME	*trans*-12,13-epoxy-octadecenoic acid	Linoleic acid
9-HODE	9-hydroxy-octadecadienoic acid	Linoleic acid
13-HODE	13-hydroxy-octadecadienoic acid	Linoleic acid
8*(E)*,12*(Z)*-10-HODE	10-hydroxy-8*(E)*,12*(Z)*-octadecadienoic acid	Linoleic acid
9*(Z)*,13*(E)*-12-HODE	12-hydroxy-9*(Z)*,13*(E)*-octadecadienoic acid	Linoleic acid
9*(Z)*,12*(Z)*-15-HODE	15-hydroxy-9*(Z)*,12*(Z)*-octadecadienoic acid	Linoleic acid
9*(Z)*,12*(Z)*-17-HODE	17-hydroxy-9*(Z)*,12*(Z)*-octadecadienoic acid	Linoleic acid
9*(Z)*,12*(Z)*-18-HODE	18-hydroxy-9*(Z)*,12*(Z)*-octadecadienoic acid	Linoleic acid
9*(Z)*-13-HOME	13-hydroxy-9*(Z)*-octadecenoic acid	Linoleic acid
9-HODA	9-hydroxy-octadecanoic acid	Linoleic acid
10-HODA	10-hydroxy-octadecanoic acid	Linoleic acid
12*(Z)*-10-HOME	10-hydroxy-12*(Z)*-octadecenoic acid	Linoleic acid
cis-9(10)-EpOME	*cis*-9(10)-epoxy-octadecenoic acid	Linoleic acid
threo-9,10-DiHOME	9,10-dihydroxy-octadecenoic acid	Linoleic acid
erythro-12,13-DiHOME	12,13-dihydroxy-octadecenoic acid	Linoleic acid
threo-12,13-DiHOME	12,13-dihydroxy-octadecenoic acid	Linoleic acid
9,12,13-TriHOME	9,12,13-trihydroxy-octadecenoic acid	Linoleic acid
threo-9,10-DiHODA	9,10-dihydroxy-octadecanoic acid	Oleic acid
cis-15(16)-EpODE	*cis*-15(16)-epoxy-octadecadienoic acid	α-Linolenic acid
9-HOTrE	9-hydroxy-octadecatrienoic acid	α-Linolenic acid
12-HOTrE	12-hydroxy-octadecatrienoic acid	α-Linolenic acid
13-HOTrE	13-hydroxy-octadecatrienoic acid	α-Linolenic acid
15-HOTrE	15-hydroxy-octadecatrienoic acid	α-Linolenic acid
16-HOTrE	16-hydroxy-octadecatrienoic acid	α-Linolenic acid
17-HOTrE	17-hydroxy-octadecatrienoic acid	α-Linolenic acid
18-HOTrE	18-hydroxy-octadecatrienoic acid	α-Linolenic acid
9*(Z)*,15*(Z)*-13-HODE	13-hydroxy-9*(Z)*,15*(Z)*-octadecadienoic acid	α-Linolenic acid
12*(Z)*,15*(Z)*-10-HODE	10-hydroxy-12*(Z)*,15*(Z)*-octadecadienoic acid	α-Linolenic acid
erythro-9,10-DiHODE	9,10-dihydroxy-octadecadienoic acid	α-Linolenic acid
threo-9,10-DiHODE	9,10-dihydroxy-octadecadienoic acid	α-Linolenic acid
erythro-12,13-DiHODE	12,13-dihydroxy-octadecadienoic acid	α-Linolenic acid
threo-12,13-DiHODE	12,13-dihydroxy-octadecadienoic acid	α-Linolenic acid
erythro-15,16-DiHODE	15,16-dihydroxy-octadecadienoic acid	α-Linolenic acid

threo-15,16-DiHODE	15,16-dihydroxy-octadecadienoic acid	α-Linolenic acid
9-F1t-PhytoP-ALA	(E,9R)-11-[(1S,2R,3R,5S)-2-ethyl-3,5-dihydroxycyclopentyl]-9-hydroxyundec-10-enoic acid	α-Linolenic acid

Table S2 – LC-MS/MS MRM transitions of oxylipins

Oxylipin	Retention time (min)	Precursor *m/z*	Product *m/z*	Method
9-F1t-PhytoP-ALA	1.3	327.3	171.3	Octadecanoids (Quaranta et al.)
9,12,13-TriHOME	3.0	329.2	211.1	Octadecanoids (Quaranta et al.)
erythro-15,16-DiHODE	4.9	311.2	223.1	Octadecanoids (Quaranta et al.)
erythro-9,10-DiHODE	5.0	311.2	201.1	Octadecanoids (Quaranta et al.)
erythro-12,13-DiHODE	5.0	311.2	213.1	Octadecanoids (Quaranta et al.)
threo-15,16-DiHODE	5.2	311.2	223.1	Octadecanoids (Quaranta et al.)
threo-9,10-DiHODE	5.3	311.2	201.1	Octadecanoids (Quaranta et al.)
threo-12,13-DiHODE	5.3	311.2	213.1	Octadecanoids (Quaranta et al.)
erythro-12,13-DiHOME	5.6	313.2	183.1	Octadecanoids (Quaranta et al.)
threo-12,13-DiHOME	6.0	313.2	183.1	Octadecanoids (Quaranta et al.)
threo-9,10-DiHOME	6.3	313.2	201.1	Octadecanoids (Quaranta et al.)
17-HOTrE	6.7	293.2	249.2	Octadecanoids (Salamin et al.)
18-HOTrE	6.8	293.2	263.2	Octadecanoids (Quaranta et al.)
15-HOTrE	6.9	293.2	223.2	Octadecanoids (Salamin et al.)
12-HOTrE	7.0	293.2	211.1	Octadecanoids (Salamin et al.)
16-HOTrE	7.0	293.2	235.2	Octadecanoids (Salamin et al.)
9-HOTrE	7.0	293.2	171.1	Octadecanoids (Quaranta et al.)
EKODE	7.1	309.2	209.1	Octadecanoids (Quaranta et al.)
13-HOTrE	7.1	293.2	195.1	Octadecanoids (Quaranta et al.)
9*(Z)*,12*(Z)*-17-HODE	7.2	295.2	251.2	Octadecanoids (Salamin et al.)
threo-9,10-DiHODA	7.3	315.2	171.1	Octadecanoids (Quaranta et al.)
9*(Z)*,12*(Z)*-18-HODE	7.4	295.2	265.2	Octadecanoids (Quaranta et al.)
9*(Z)*,12*(Z)*-15-HODE	7.5	295.2	223.1	Octadecanoids (Quaranta et al.)
12*(Z)*,15*(Z)*-10-HODE	7.6	295.2	185.1	Octadecanoids (Quaranta et al.)
9*(Z)*,15*(Z)*-13-HODE	7.7	295.2	225.1	Octadecanoids (Quaranta et al.)
9*(Z)*,13*(E)*-12-HODE	7.9	295.2	183.1	Octadecanoids (Quaranta et al.)
13-HODE	8.0	295.2	195.1	Octadecanoids (Quaranta et al.)
9-HODE	8.1	295.2	171.1	Octadecanoids (Quaranta et al.)
8*(E)*,12*(Z)*-10-HODE	8.1	295.2	183.1	Octadecanoids (Quaranta et al.)
cis-15(16)-EpODE	8.2	293.2	235.1	Octadecanoids (Quaranta et al.)
9-KODE	8.6	293.2	185.1	Octadecanoids (Quaranta et al.)
9*(Z)*-13-HOME	8.6	297.2	113.1	Octadecanoids (Quaranta et al.)
12*(Z)*-10-HOME	8.7	297.2	185.1	Octadecanoids (Quaranta et al.)
9*(Z)*-13-KOME	9.0	295.2	113.1	Octadecanoids (Quaranta et al.)
cis-12(13)-EpOME	9.3	295.2	195.1	Octadecanoids (Quaranta et al.)
trans-12(13)-EpOME	9.5	295.2	195.1	Octadecanoids (Quaranta et al.)
cis-9(10)-EpOME	9.5	295.2	171.1	Octadecanoids (Quaranta et al.)

Oxylipin	Retention time (min)	Precursor *m/z*	Product *m/z*	Method
10-HODA	10.0	299.2	141.2	Octadecanoids (Salamin et al.)
9-HODA	10.3	299.2	155.1	Octadecanoids (Salamin et al.)
6-keto-PGF α	1.5	369.2	163.1	Eicosanoids (Kolmert et al.)
TXB_1	1.9	371.2	171.1	Eicosanoids (Kolmert et al.)
TXB_2	2.1	369.2	169.1	Eicosanoids (Kolmert et al.)
17,18-DiHETE	5.9	335.2	247.1	Eicosanoids (Kolmert et al.)
19,20-DiHDPA	7.2	361.2	229.1	Eicosanoids (Kolmert et al.)
14,15-DiHETrE	7.2	337.2	207.1	Eicosanoids (Kolmert et al.)
12-HHTrE	7.3	279.2	179.1	Eicosanoids (Kolmert et al.)
11,12-DiHETrE	7.6	337.2	167.1	Eicosanoids (Kolmert et al.)
8,9-DiHETrE	8.1	337.2	127.1	Eicosanoids (Kolmert et al.)
12-HEPE	8.7	317.2	179.1	Eicosanoids (Kolmert et al.)
15-HETE	9.4	319.2	175.1	Eicosanoids (Kolmert et al.)
11-HETE	9.7	319.2	167.1	Eicosanoids (Kolmert et al.)
12-HETE	9.9	319.2	179.1	Eicosanoids (Kolmert et al.)
5-HETE	10.4	319.2	115.1	Eicosanoids (Kolmert et al.)

Table shows MRM transitions of 32 octadecanoids previously reported by Quaranta et al., 13 eicosanoids (and a single docosanoid) reported by Kolmert et al, and 7 new octadecanoids from Salamin et al. (unpublished work) used for LC-MS/MS analysis in negative ion mode – each referring to the $[M-H]^-$ precursor ion. Oxylipin nomenclature is as reported in Table S1.

Quaranta, A.; Zöhrer, B.; Revol-Cavalier, J.; Benkestock, K.; Balas, L.; Oger, C.; Keyes, G. S.; Wheelock, Å. M.; Durand, T.; Galano, J.-M.; Ramsden, C. E.; Hamberg, M.; Wheelock, C. E. Development of a Chiral Supercritical Fluid Chromatography–Tandem Mass Spectrometry and Reversed-Phase Liquid Chromatography–Tandem Mass Spectrometry Platform for the Quantitative Metabolic Profiling of Octadecanoid Oxylipins. *Anal. Chem.* **2022**, *94* (42), 14618–14626.

Kolmert, J.; Fauland, A.; Fuchs, D.; Säfholm, J.; Gómez, C.; Adner, M.; Dahlén, S.-E.; Wheelock, C. E. Lipid Mediator Quantification in Isolated Human and Guinea Pig Airways: An Expanded Approach for Respiratory Research. *Anal. Chem.* **2018**, *90* (17), 10239–10248.

Table S3 – Internal standards and their final concentration in the LC-MS/MS internal standard mixture

Internal standard	Concentration (ng/ml)	Method
d11-11(12)-EpETrE	37.5	Eicosanoids (Kolmert et al.)
d11-14,15-DiHETrE	20	Eicosanoids (Kolmert et al.)
d5-LTE4	40	Eicosanoids (Kolmert et al.)
d4-6-keto-PGF1a	100	Eicosanoids (Kolmert et al.)
d4-9(10)-DiHOME	32	Eicosanoids (Kolmert et al.)
d4-9(10)-EpOME	70	Eicosanoids (Kolmert et al.)
d4-9-HODE	20	Eicosanoids (Kolmert et al.)
d4-LTB4	75	Eicosanoids (Kolmert et al.)
d4-PGB2	40	Eicosanoids (Kolmert et al.)
d4-PGD2	68	Eicosanoids (Kolmert et al.)
d4-PGE2	20	Eicosanoids (Kolmert et al.)
d4-TXB2	42	Eicosanoids (Kolmert et al.)
d6-20-HETE	100	Eicosanoids (Kolmert et al.)
d7-5-oxo-ETE	100	Eicosanoids (Kolmert et al.)
d8-15-HETE	32	Eicosanoids (Kolmert et al.)
d8-5-HETE	32	Eicosanoids (Kolmert et al.)
d11-11,12-DiHETrE	20	Eicosanoids (Kolmert et al.)
d11-5(6)-EpETrE	40	Eicosanoids (Kolmert et al.)
d11-5-iPF2α-VI	32	Eicosanoids (Kolmert et al.)
d11-8(9)-EpETrE	90	Eicosanoids (Kolmert et al.)
d11-8,9-DiHETrE	32	Eicosanoids (Kolmert et al.)
d3-13-KODE	30	Eicosanoids (Kolmert et al.)
d4-11-keto TXB2	40	Eicosanoids (Kolmert et al.)
d4-12,13-DiHOME	35	Eicosanoids (Kolmert et al.)
d4-13-HODE	30	Eicosanoids (Kolmert et al.)
d4-13,14-dh-15-keto PGE2	42	Eicosanoids (Kolmert et al.)
d4-8-epiPGF2α	32	Eicosanoids (Kolmert et al.)
5-iPF2α-VI-D11/d4-iPF2α-VI	32	Eicosanoids (Kolmert et al.)
d4-PGD1	125	Eicosanoids (Kolmert et al.)
d5-LXA4	32	Eicosanoids (Kolmert et al.)
d5-Resolvin D2	60	Eicosanoids (Kolmert et al.)
d8-12-HETE	32	Eicosanoids (Kolmert et al.)
d5-LTC4	32	Eicosanoids (Kolmert et al.)
d5-LTD4	32	Eicosanoids (Kolmert et al.)
d5-DHA	8	Eicosanoids (Kolmert et al.)
d5-EPA	12	Eicosanoids (Kolmert et al.)
d4-11-β-PGF2a	75	Eicosanoids (Kolmert et al.)
d4-8-iso-PGE2	60	Eicosanoids (Kolmert et al.)
d4-PGF2a	60	Eicosanoids (Kolmert et al.)
d4-PGJ2	65	Eicosanoids (Kolmert et al.)

Internal standard	Concentration (ng/ml)	Method
d5-Maresin 1	60	Eicosanoids (Kolmert et al.)
d4-12(13)-EpOME	70	Eicosanoids (Kolmert et al.)
d3-9-oxo-ODE	35	Eicosanoids (Kolmert et al.)
d5-13-HOTrE	50	Eicosanoids (Kolmert et al.)
d5-17S-HDHA /HDoHE	37.5	Eicosanoids (Kolmert et al.)
13-KODE-d3	100	Octadecanoids (Quaranta et al.)
9(S)-HODE-d4	100	Octadecanoids (Quaranta et al.)
13(S)-HODE-d4	100	Octadecanoids (Quaranta et al.)
9(10)-EpOME-d4	125	Octadecanoids (Quaranta et al.)
12(13-EpOME-d4	300	Octadecanoids (Quaranta et al.)
9,10-DiHOME-d4	100	Octadecanoids (Quaranta et al.)
12,13-DiHOME-d4	100	Octadecanoids (Quaranta et al.)
9,12,13-(13C3)-TriHOME	50	Octadecanoids (Quaranta et al.)
13-OH-9,10-EpOME-d5	125	Octadecanoids (Quaranta et al.)
16-F1t-PhytoP-C19	100	Octadecanoids (Quaranta et al.)
12(Z)-10-KOME-d5	200	Octadecanoids (Quaranta et al.)
12(Z)-10-HOME-d5	100	Octadecanoids (Quaranta et al.)
13-HOTrE-d5	100	Octadecanoids (Quaranta et al.)

Table shows the internal standards used for normalization of oxylipin response in the octadecanoid and eicosanoid methods as well as their combined final concentrations prior to spiking in the LC-MS/MS study samples. Oxylipin nomenclature is as reported in Table S1.

Kolmert, J.; Fauland, A.; Fuchs, D.; Säfholm, J.; Gómez, C.; Adner, M.; Dahlén, S.-E.; Wheelock, C. E. Lipid Mediator Quantification in Isolated Human and Guinea Pig Airways: An Expanded Approach for Respiratory Research. *Anal. Chem.* **2018**, *90* (17), 10239–10248.

Quaranta, A.; Zöhrer, B.; Revol-Cavalier, J.; Benkestock, K.; Balas, L.; Oger, C.; Keyes, G. S.; Wheelock, Å. M.; Durand, T.; Galano, J.-M.; Ramsden, C. E.; Hamberg, M.; Wheelock, C. E. Development of a Chiral Supercritical Fluid Chromatography–Tandem Mass Spectrometry and Reversed-Phase Liquid Chromatography–Tandem Mass Spectrometry Platform for the Quantitative Metabolic Profiling of Octadecanoid Oxylipins. *Anal. Chem.* **2022**, *94* (42), 14618–14626.

Table S4 – DESI-MRM transitions

Compound name	Precursor Ion	Product Ion	Cone (V)	Collision (eV)	Structure
9-HODE	295.2	171.1	25	19	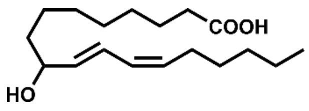
16-HOTrE	293.2	235.2	25	13	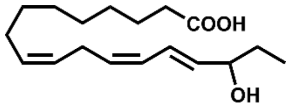
12,13-DiHOME	313.2	183.1	25	20	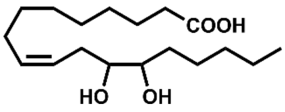
11-HETE	319.2	167.1	27	15	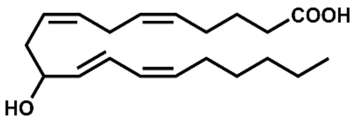
12-HETE	319.2	179.1	28	14	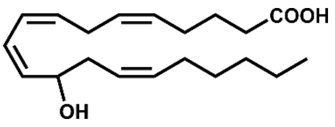
9-HETE*	319.2	179.1	28	14	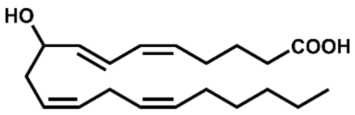
TXB_2	369.2	169.1	26	15	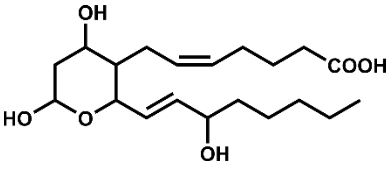
Arachidonic acid	303.3	259.2	20	12	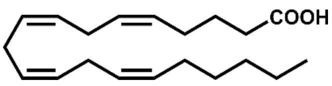

Table shows MRM transitions used for DESI-MS/MS analysis of oxylipins in negative ion mode – each referring to the $[M\text{-}H]^-$ precursor ion. Oxylipin nomenclature is as reported in Table S1.

*The 9-HETE shares the same transition as 12-HETE; however, the orthogonal LC-MS/MS analysis reported that the 9-HETE was <LLOD in the guinea pig lung tissues. We therefore determined that the transition was specific to 12-HETE in this tissue.

Table S5 – LC-MS/MS data

Oxylipin	Parenchyma (pg/mg)	Airways (pg/mg)	PC1	PC2	P-value	Fold change
9-KODE	5.66E+00	6.83E-01	-6.84E-02	-2.10E-03	5.44E-02	1.21E-01
9*(Z)*-13-KOME	3.60E+00	1.66E+01	1.02E-01	2.80E-02	3.89E-02	4.60E+00
cis-15(16)-EpODE	1.35E+01	2.60E+00	-1.17E-01	-2.77E-03	3.01E-02	1.93E-01
cis-9(10)-EpOME	1.73E+00	<LLOD	ND	ND	ND	ND
cis-12(13)-EpOME	3.70E+00	<LLOD	ND	ND	ND	ND
trans-12(13)-EpOME	2.66E+00	3.08E+00	-1.20E-02	2.16E-03	4.44E-01	1.16E+00
9-HOTrE	6.68E-01	5.07E+00	6.64E-02	1.07E-02	3.15E-02	7.58E+00
12-HOTrE	<LLOD	3.40E+00	ND	ND	ND	ND
13-HOTrE	<LLOD	1.40E+01	ND	ND	ND	ND
15-HOTrE	6.71E-01	4.51E+00	5.42E-02	1.13E-02	1.12E-01	6.71E+00
16-HOTrE	1.27E+01	2.69E+01	1.13E-01	4.60E-02	2.04E-02	2.12E+00
17-HOTrE	7.75E+01	2.19E+01	-2.82E-01	5.03E-02	1.47E-02	2.83E-01
18-HOTrE	1.99E+00	2.68E-01	2.10E-02	7.56E-03	3.91E-01	1.35E-01
9*(Z)*,15*(Z)*-13-HODE	8.28E+00	5.59E+01	2.28E-01	2.50E-02	6.69E-03	6.76E+00
9-HODE	2.12E+01	4.44E+01	1.55E-01	5.33E-02	4.57E-02	2.10E+00
13-HODE	3.61E+00	3.65E+01	1.94E-01	4.26E-02	3.15E-02	1.01E+01
8*(E)*,12*(Z)*-10-HODE	5.34E-01	2.67E+00	ND	ND	1.68E-01	5.00E+00
9*(Z)*,13*(E)*-12-HODE	1.19E-01	1.54E+00	1.14E-02	-2.49E-02	5.25E-02	1.29E+01
9*(Z)*,12*(Z)*-15-HODE	8.36E-01	3.44E+00	5.29E-02	8.76E-03	1.03E-02	4.12E+00
9*(Z)*,12*(Z)*-17-HODE	3.75E+01	2.26E+00	-2.11E-01	2.39E-02	2.33E-02	6.04E-02
9*(Z)*,12*(Z)*-18-HODE	2.48E+00	8.66E-01	-3.89E-02	1.51E-02	4.42E-02	3.49E-01
9*(Z)*-13-HOME	3.56E+00	3.44E+01	ND	ND	3.63E-01	9.65E+00
9-HODA	4.22E+01	6.65E+01	1.14E-01	-3.53E-01	4.71E-01	1.58E+00
10-HODA	1.91E+02	1.87E+02	3.53E-02	-8.26E-01	5.63E-01	9.80E-01
12*(Z)*-10-HOME	1.06E+01	6.64E+00	-2.54E-02	-6.68E-02	1.78E-01	6.25E-01
12*(Z)*,15*(Z)*/11*(E)*,15*(Z)*-10-HODE	4.34E+00	8.24E+00	7.45E-02	1.58E-02	4.57E-02	1.90E+00
EKODE	3.93E+00	5.40E+00	7.58E-03	-3.58E-02	6.37E-01	1.37E+00
erythro-9,10-DiHODE	4.80E-01	<LLOD	3.63E-02	-6.09E-04	3.63E-01	ND
threo-9,10-DiHODE	6.88E+00	1.98E+00	-7.97E-02	4.32E-02	6.69E-03	2.87E-01
erythro-12,13-DiHODE	1.03E+01	<LLOD	9.70E-02	2.52E-02	ND	ND
threo-12,13-DiHODE	6.60E+00	<LLOD	3.93E-02	5.69E-02	ND	ND
erythro-15,16-DiHODE	6.21E+00	7.92E-01	-7.75E-02	-3.76E-03	2.51E-02	1.28E-01
threo-15,16-DiHODE	5.42E+01	1.23E+01	-2.47E-01	7.69E-02	6.69E-03	2.26E-01
threo-9,10-DiHOME	1.56E+01	3.38E+00	-1.29E-01	7.69E-02	6.69E-03	2.17E-01
erythro-12,13-DiHOME	3.78E+00	<LLOD	-5.51E-03	2.94E-02	ND	ND
threo-12,13-DiHOME	3.79E+01	8.65E+00	-1.94E-01	1.19E-01	6.69E-03	2.28E-01
threo-9,10-DiHODA	1.73E+02	1.26E+01	-4.84E-01	1.42E-01	1.01E-02	7.25E-02
9,12,13-TriHOME	3.76E+00	6.82E+00	4.32E-02	8.98E-03	5.25E-02	1.81E+00
9-F1t-PhytoP-ALA	3.10E+00	<LLOD	ND	ND	ND	ND
19,20-DiHDPA	1.17E+01	9.17E-01	-4.85E-02	7.78E-02	6.72E-02	7.83E-02
17,18-DiHETE	5.95E+01	<LLOD	ND	ND	ND	ND

Oxylipin	Parenchyma (pg/mg)	Airways (pg/mg)	PC1	PC2	P-value	Fold change
8,9-DiHETrE	3.18E+00	4.61E+00	2.26E-02	5.25E-02	5.40E-01	1.45E+00
11,12-DiHETrE	5.37E-01	4.46E-01	ND	ND	1.00E+00	8.32E-01
14,15-DiHETrE	4.56E+00	2.77E+00	-3.39E-02	6.47E-02	3.82E-01	6.08E-01
12-HEPE	4.02E+00	1.59E+01	1.27E-01	7.03E-02	1.68E-01	3.95E+00
5-HETE	1.91E+00	9.13E+00	9.65E-02	6.15E-02	5.25E-02	4.79E+00
11-HETE	1.01E+01	1.68E+01	8.80E-02	1.09E-01	1.63E-01	1.67E+00
12-HETE	2.94E+01	1.15E+02	3.64E-01	2.27E-01	4.42E-02	3.90E+00
15-HETE	2.97E+00	9.40E+00	1.15E-01	7.23E-02	1.84E-01	3.17E+00
12-HHTrE	1.87E+00	1.71E+01	1.35E-01	7.59E-02	5.25E-02	9.10E+00
6-keto-PGF1a	4.58E+00	4.01E+01	2.20E-01	7.94E-02	3.89E-02	8.74E+00
TXB1	1.97E-01	5.37E-01	ND	ND	1.00E+00	2.72E+00
TXB2	2.25E+01	6.67E+01	2.51E-01	1.39E-01	1.95E-02	2.96E+00

Table shows the concentration in each tissue type (pg/mg), principal component analysis eigenvalues (PC1 and PC2), fold change airways:parenchyma (FC) and Benjamini and Hochberg False Discovery Rate (FDR) corrected p-value). Oxylipin nomenclature is as reported in Table S1.

<LLOD in either the parenchyma or airways headers indicates that the oxylipin was not detected due to it being below the lower limit of detection in this tissue type.

ND in PC1 and PC2 indicates that the peak matrix processing for multivariate analysis removed the oxylipin (due to sparsity across the sample space)

ND for *P*-value or Fold Change indicates that the lipid was <LLOD in either one or both tissue types.

Table S6 – Calibration curve preparation of 12,13-DiHOME for quantification

Calibration Level	Replicate	Amount Deposited (pg)	Pixel count	pg/pixel	DESI-MRM response
L1	1	0	2505	0.00E+00	2.83E+03
L2	1	0.5	2908	1.72E-04	3.32E+03
L3	1	1	3057	3.27E-04	3.46E+03
L4	1	5	2673	1.87E-03	1.16E+04
L5	1	10	2438	4.10E-03	2.32E+04
L6	1	25	1847	1.35E-02	4.81E+04
L7	1	NA	NA	NA	NA
L8	1	400	2803	1.43E-01	3.83E+05
R^2 = 0.99<br>response = 2.71E+06 [pg/pixel] + 1.63E+03					
L1	2	0	1937	0.00E+00	2.27E+03
L2	2	0.5	2923	1.71E-04	2.91E+03
L3	2	1	3637	2.75E-04	3.16E+03
L4	2	5	2717	1.84E-03	1.05E+04
L5	2	10	2730	3.66E-03	1.77E+04
L6	2	25	1637	1.53E-02	5.15E+04
L7	2	150	2952	5.08E-02	1.64E+05
L8	2	400	2586	1.55E-01	3.61E+05
R^2 = 0.97<br>response = 2.53E+06 [pg/pixel] + 5.82E+03					
L1	3	0	3969	0.00E+00	2.82E+03
L2	3	0.5	3502	1.43E-04	3.50E+03
L3	3	1	3597	2.78E-04	4.54E+03
L4	3	5	3641	1.37E-03	1.21E+04
L5	3	10	3584	2.79E-03	2.00E+04
L6	3	25	2033	1.23E-02	4.69E+04
L7	3	150	2787	5.38E-02	1.80E+05
L8	3	400	3058	1.31E-01	4.39E+05
R^2 = 0.99<br>response = 3.33E+06 [pg/pixel] + 1.90E+02					
**R^2 = 0.97**<br>**response = 2.85E+06 [pg/pixel] + 3.57E+03**					

Table shows the amount of 12,13-DiHOME deposited onto guinea pig liver for each replicate of each calibration level and the number of pixels the standard was distributed over – with the pg/pixel denoting the theoretical concentration. DESI-MRM response refers to the mean pixel response across the pixels in each calibration spot. For each replicate, the equation of the linear fit (DESI-MRM response *vs* concentration) is provided and the R^2, with details of the overall fit provided at the bottom of the table.

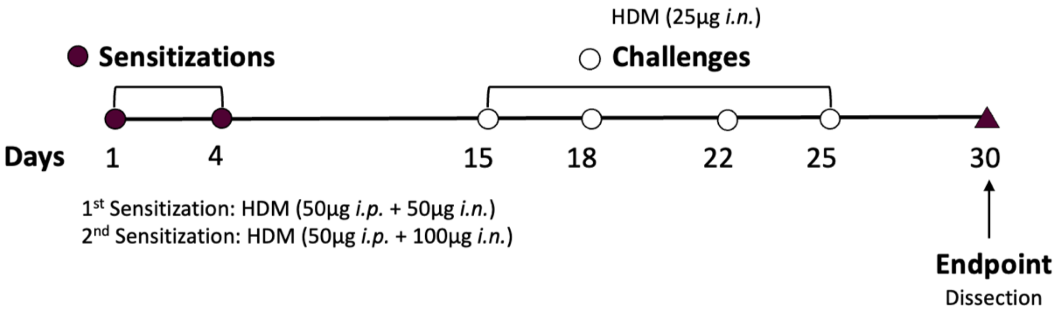

Figure S1 – *In vivo* guinea pig model. Schematic depicting the house dust mite (HDM) sensitization and challenge protocol for development of the guinea pig asthma model. *i.n.*=intranasal. *i.p.*=intraperitoneal.

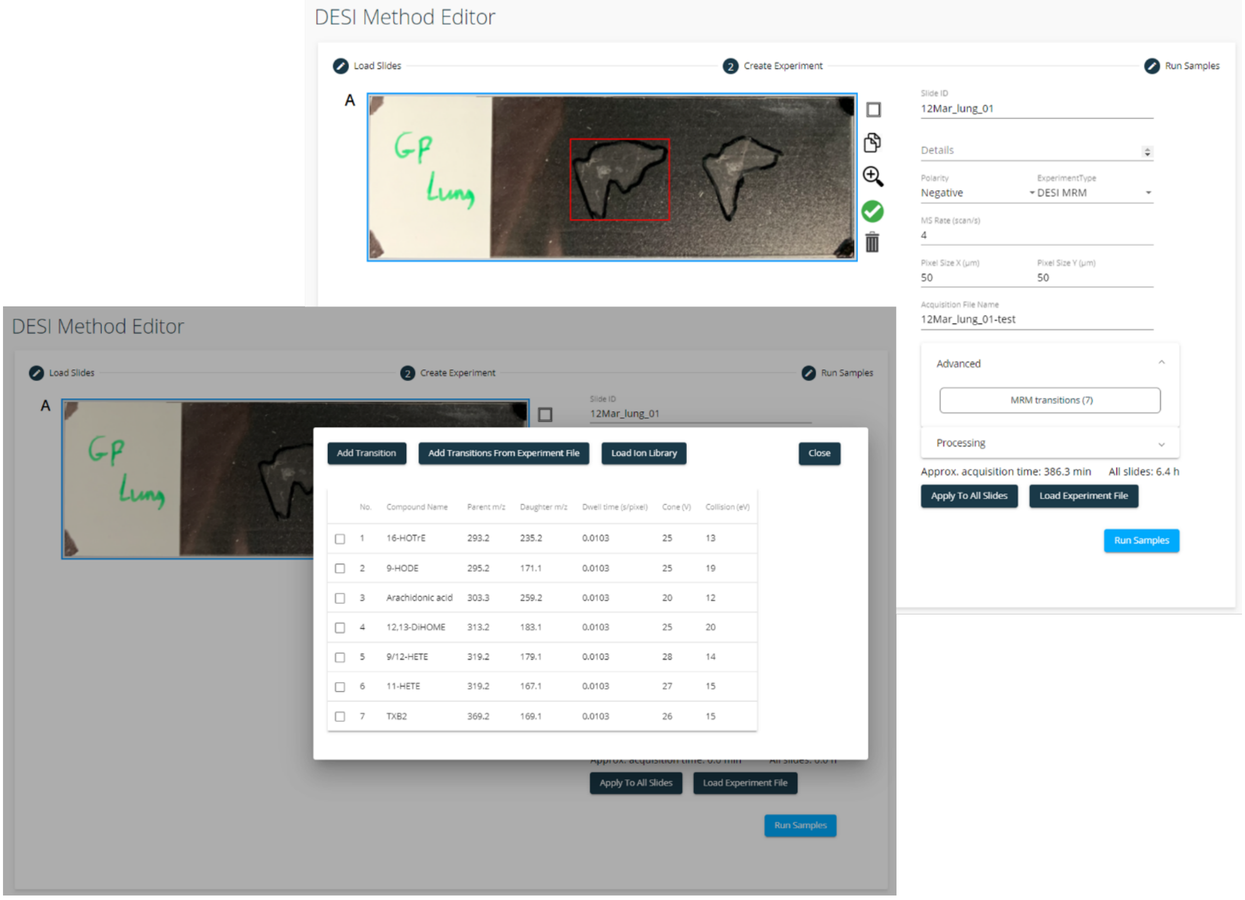

Figure S2 – DESI-MRM experiment set-up. Details of how-to set-up a DESI-MRM experiment using DESI Method Editor within HDImaging v1.7 (Waters). Depicted here are 6 oxylipin MRM transitions (as well as arachidonic acid) to be detected from guinea pig lung tissue at 50 μm^2 pixels sizes at 4 Hz in negative ion mode.

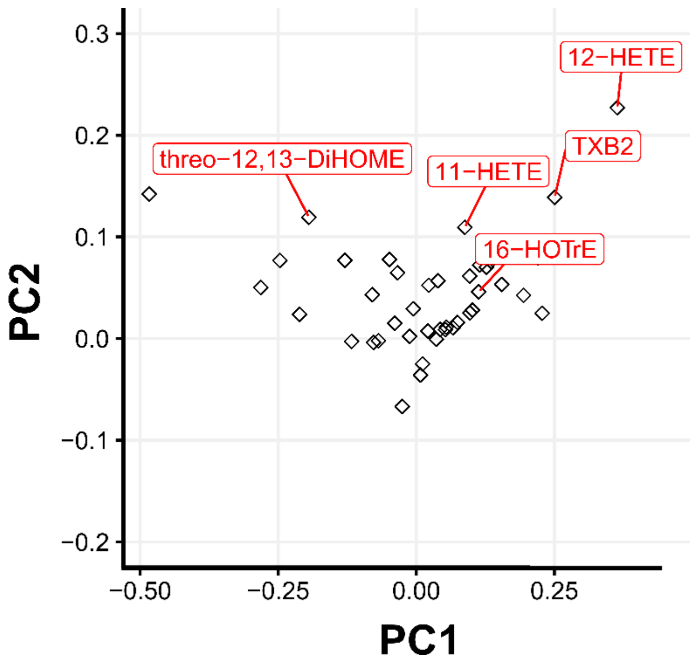

Figure S3 – LC-MS/MS analysis of oxylipin extracts from isolated guinea pig lung tissue. Principal component analysis (PCA) loadings plot of all 53 quantified oxylipins, with those selected for DESI-MRM analysis labeled. Oxylipin nomenclature is as reported in Table S1.

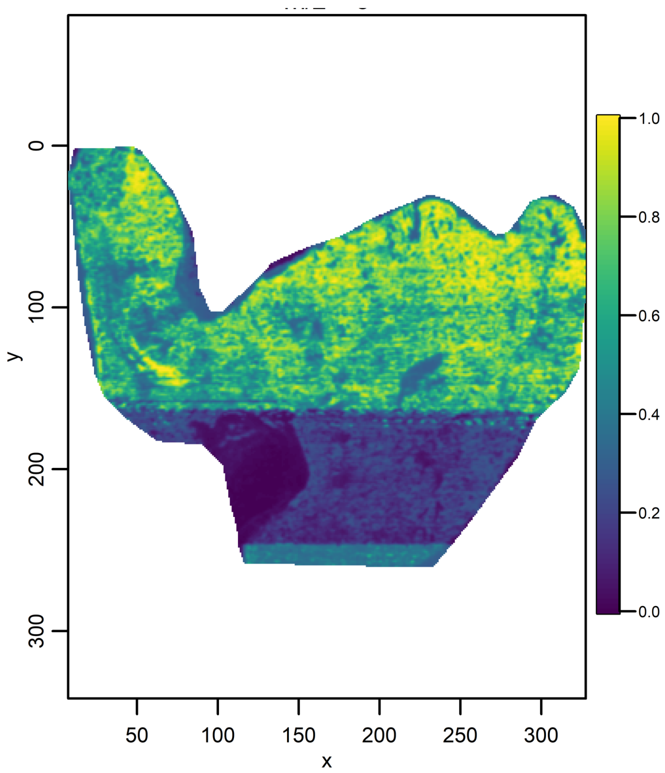

Figure S4 – DESI-MRM imaging of SM(d18:1/16:0) in guinea pig lung tissue. Shows the image artefact caused by a drop in back pressure of the binary solvent manager delivering the DESI solvent discovered during method development.

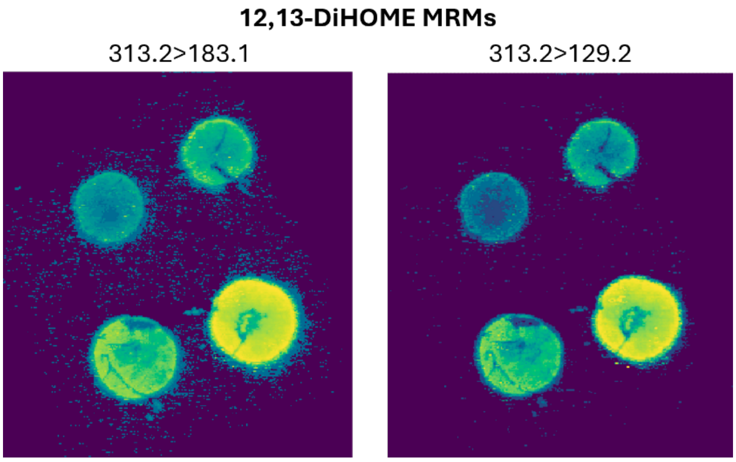

Figure S5 – DESI-MRM imaging of two transition relating to the 12,13-DiHOME standard spotted on guinea pig liver sections. Ion images of 12,13-DiHOME spotted onto liver sections at 10, 25, 150 and 400 pg per spot. The ion image on left is for the 313.2>183.1 transition and the 313.2>129.2 transition is shown on the right. The Pearson's correlation between the MRM transitions was 0.97.

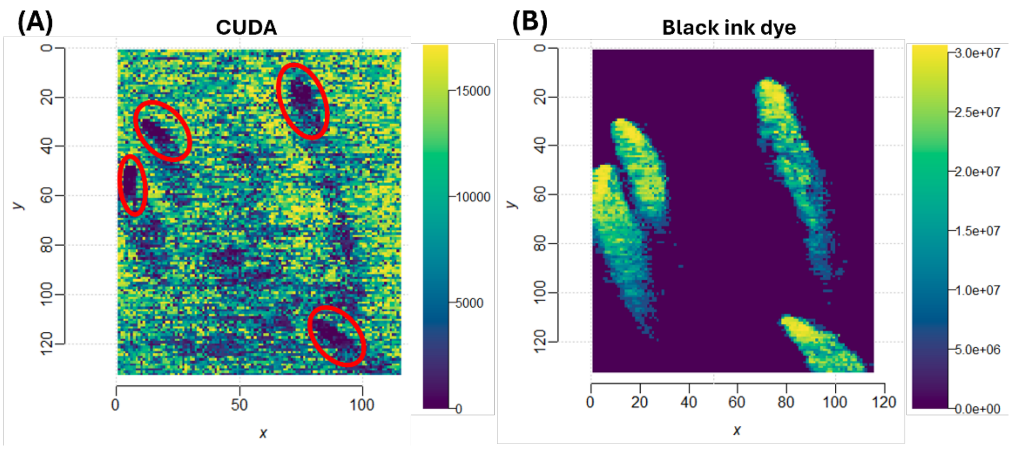

Figure S6 – DESI-MRM imaging of the technical internal standard and black ink across a blank slide. A glass slide was marked with black ink and the corresponding ion images were acquired for the technical internal standard, 12-[[(cyclohexylamino)carbonyl]amino]-dodecanoic acid (CUDA), from the DESI spray at 5 ng/mL. The transition 339.26>214.15 was used for CUDA (on the left) and the transition 666.06>666.06 was used for the black ink (on the right). The black ink had an intensity that was 2000 times greater. The red circles highlight ion suppression of CUDA caused by the black ink.

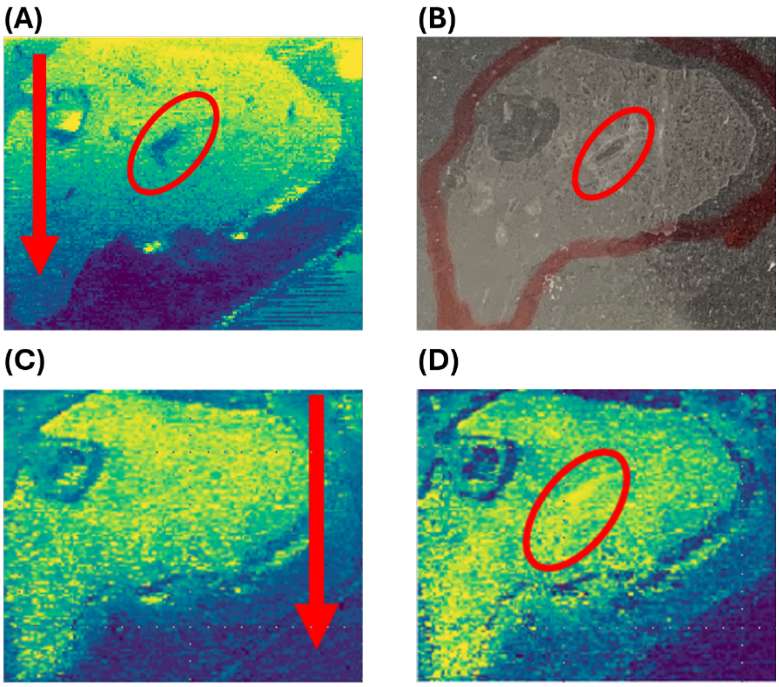

Figure S7 – DESI-MRM imaging of hexose in guinea pig lung tissue before and after correction to the technical internal standard. (A) Ion image of the technical internal standard, 12-[[(cyclohexylamino)carbonyl]amino]-dodecanoic acid (CUDA) with transition 339.26>214.15, from the DESI spray at 5 ng/mL. An overall drop in signal in the y-direction is observed (red arrow), with a local drop to ~0 intensity circled in red, which corresponds to an airway (*i.e.,* absence of tissue) as highlighted in the optical image in panel B. (B) Photo of the lung tissue section on a glass slide with the airway circled in red indicating the region in which CUDA signal intensity dropped. (C) Non-normalized ion image of the non-specific 6-carbon hexose sugar transition (215.1>35.0). (D) Ion image of the non-specific 6-carbon hexose sugar transition (215.1>35.0) normalized to CUDA intensity. The red circle highlights the region with an absence of tissue (*i.e.,* airways) where normalization has created an artefact in the data.

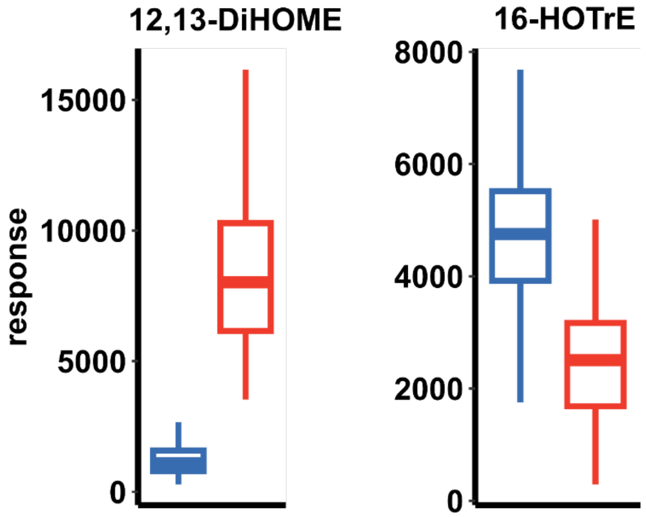

Figure S8 – DESI-MRM imaging of selected oxylipins in guinea pig lung tissue. Boxplots of 12,13-DiHOME and 16-HOTrE based on their DESI-MRM response in 50 pixels in both guinea pig airways (blue) and lung parenchyma (red).

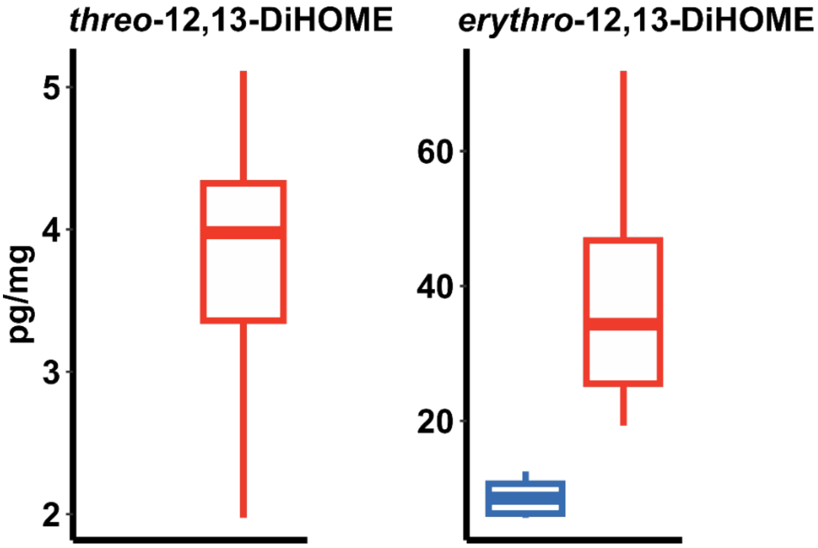

Figure S9 – LC-MS/MS analysis of oxylipin extracts from isolated guinea pig lung tissue. Boxplots showing the concentration of the *threo*- and *erythro*-12,13-DiHOME stereoisomers distinguished by LC-MS/MS analysis of C48/80-exposed guinea pig airways (blue) and lung parenchyma (red).

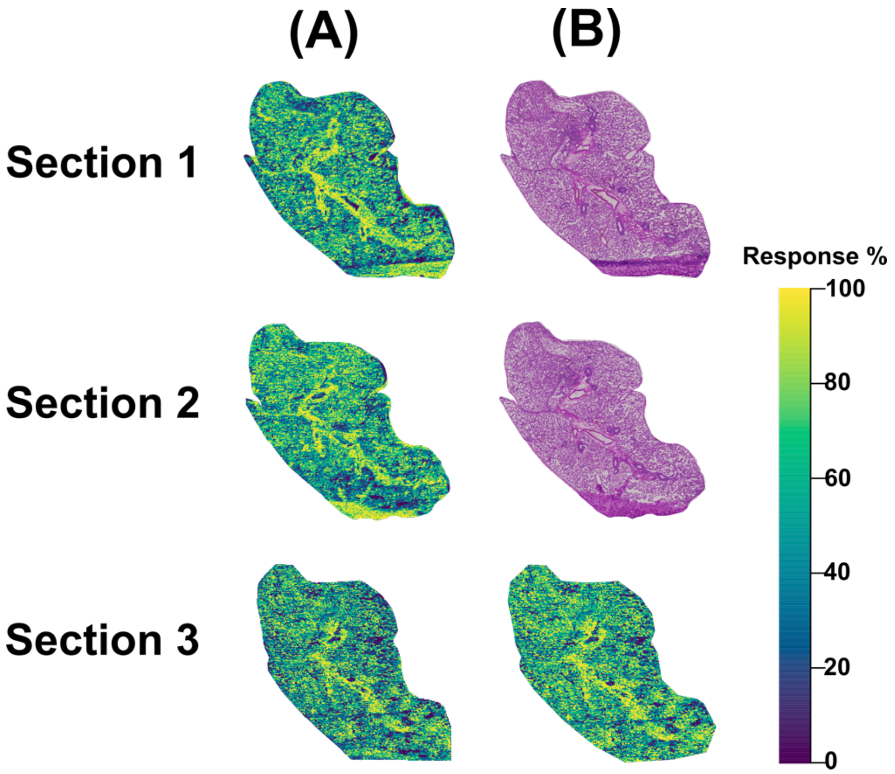

Figure S10 – DESI-MRM imaging of 16-HOTrE across sequential sections and repeat acquisitions. (A) Ion images of 16-HOTrE from 3 sequential sections of the same guinea pig lung. Section 3 was analyzed 6 months after Sections 1 and 2 (with slide stored at -80°C). (B) Associated H&E stains from Sections 1 and 2, followed by duplicate DESI-MRM analysis of 16-HOTrE from Section 3 acquired 2 days apart.

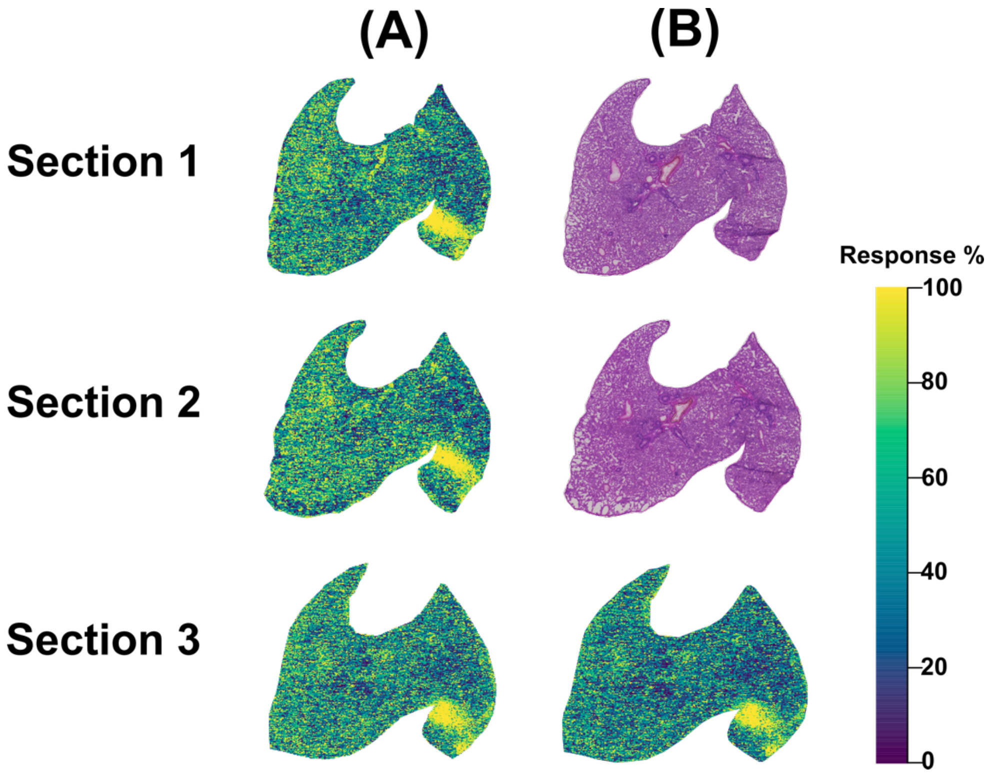

Figure S11 – DESI-MRM imaging of TXB_2 across sequential sections and repeat acquisitions. (A) Ion images of TXB_2 from 3 sequential sections of the same guinea pig lung. Section 3 was analyzed 6 months after Sections 1 and 2 (with slide stored at -80°C). (B) Associated H&E stains from Sections 1 and 2, followed by duplicate DESI-MRM analysis of TXB_2 from Section 3 acquired 2 days apart.
